# Oral microflora and pregnancy: a systematic review and meta-analysis

**DOI:** 10.1038/s41598-021-96495-1

**Published:** 2021-08-19

**Authors:** Hoonji Jang, Alexa Patoine, Tong Tong Wu, Daniel A. Castillo, Jin Xiao

**Affiliations:** 1 Eastman Institute for Oral Health, University of Rochester Medical Center, Rochester, NY USA; 2 Department of Biostatistics and Computational Biology, University of Rochester Medical Center, Rochester, USA; 3 Miner Library, University of Rochester Medical Center, Rochester, NY USA; 4 Perinatal Oral Health, Eastman Institute for Oral Health, University of Rochester, 625 Elmwood Ave, Rochester, 14620 USA

**Keywords:** Dental caries, Oral microbiology, Oral diseases

## Abstract

Understanding changes in oral flora during pregnancy, its association to maternal health, and its implications to birth outcomes is essential. We searched PubMed, Embase, Web of Science, and Cochrane Library in May 2020 (updated search in April and June 2021), and conducted a systematic review and meta-analyses to assess the followings: (1) oral microflora changes throughout pregnancy, (2) association between oral microorganisms during pregnancy and maternal oral/systemic conditions, and (3) implications of oral microorganisms during pregnancy on birth outcomes. From 3983 records, 78 studies were included for qualitative assessment, and 13 studies were included in meta-analysis. The oral microflora remains relatively stable during pregnancy; however, pregnancy was associated with distinct composition/abundance of oral microorganisms when compared to postpartum/non-pregnant status. Oral microflora during pregnancy appears to be influenced by oral and systemic conditions (e.g. gestational diabetes mellitus, pre-eclampsia, etc.). Prenatal dental care reduced the carriage of oral pathogens (e.g. *Streptococcus mutans*). The *Porphyromonas gingivalis* in subgingival plaque was more abundant in women with preterm birth. Given the results from meta-analyses were inconclusive since limited studies reported outcomes on the same measuring scale, more future studies are needed to elucidate the association between pregnancy oral microbiota and maternal oral/systemic health and birth outcomes.

## Introduction

Pregnancy is a unique physiological state, accompanied by temporary changes in women’s physical structure, hormone levels, metabolism and immune systems^1,2^. The changes during pregnancy are vital to maintaining the stable status of mother and fetus, however, some physiological, hormonal and dietary changes associated with pregnancy, in turn, alter the risk for oral diseases, such as periodontal disease and dental caries^3^. The delicate and complex changes during pregnancy also affect the microbial composition of various body sites of the expectant mothers^4^, including the oral cavity^2^. The oral cavity is colonized with a complex and diverse microbiome of over 700 commensals that have been identified in the Human Oral Microbiome Database (HOMD)^5^ and recently expanded HOMD (*e*HOMD), including bacterial and fungal species^6^. Given a balanced microbial flora helps to maintain stable oral and general health, alterations in the oral microbial community during pregnancy might impact maternal oral health^7,8^, birth outcomes^9^, and the infant’s oral health^10^. Therefore, understanding changes of oral flora during pregnancy, its association to maternal health, and its implications to birth outcomes is essential.

First, despite the speculated associations between oral flora and oral diseases during pregnancy, two critical questions that remain to be answered are (1) what changes in the oral microbiota occur during pregnancy; (2) whether the changes are associated with increased risk for oral diseases during pregnancy. Studies that evaluated the stability of the oral microbiome during pregnancy revealed that the composition and diversity of oral microbiome components remained stable without significant change^11,12^. However, on the contrary, some studies reported that pregnant women experienced a significant increase in *Streptococcus mutans*, a well-known culprit for dental caries^13,14^. In addition, researchers also reported an increased level of periodontal pathogens, e.g., *Aggregatibacter actinomycetemcomitans*, *Porphyromona gingivalis* and *Prevotella intermedia*, among pregnant women^15–17^. Nevertheless, comprehensive evaluations of available evidence are needed to provide conclusive consensus.

Second, a clear understanding of the association between oral microorganisms and adverse birth outcomes conveys significant health implications. A systematic review from Daalderop et al., reported an association between periodontal disease and various adverse pregnancy outcomes^18^. Women who have periodontal diseases during pregnancy are at higher risk for delivering preterm and low birth-weight infants^19–21^. In terms of oral microorganisms, researchers reported a higher level of *P. gingivalis* among women with preterm deliveries^22,23^. A higher risk of preterm delivery was also observed among pregnant women with detection of periodontal anaerobes in subgingival plaque^24^. In contrast, Costa et al. reported that the risk of preterm birth is not correlated to an increased amount of periodontopathogenic bacteria^25^. Therefore, a thorough review of all available evidence on the topic of prenatal oral microorganisms and adverse birth outcomes is critical.

Furthermore, maternal oral health is closely associated with children’s oral health, including maternal relatedness and vertical transmission of oral pathogens from mothers to infants^26^. Thus, in theory, reducing maternal oral pathogens during pregnancy is paramount, since it could potentially reduce or delay the colonization of oral pathogens in the infant’s oral cavity. Interestingly, although some studies^27,28^ demonstrated that expectant mothers who received atraumatic dental restorative treatment during pregnancy resulted in significant reductions of *S. mutans* carriage, and pregnant women who received periodontal treatment (scaling and root planning) had a lowered periodontal pathogen level, a study from Jaramillo et al., failed to indicate decreased periodontal bacteria in pregnant women following periodontal treatment^29^.

Therefore, this study aims to comprehensively review the literature on oral microorganisms and pregnancy. We are focusing on analyzing the evidence on the following subcategories: (1) oral microbial community changes throughout pregnancy, including changes of key oral pathogens, the abundance, and diversity of the oral fungal and bacterial community; (2) association between oral microorganisms during pregnancy and maternal oral/systemic diseases; (3) implications of oral microorganisms during pregnancy on adverse birth outcomes.

## Methods

This systematic review followed the PRISMA guidelines^30^, the protocol was registered for in the PROSPERO (CRD42021246545) (https://www.crd.york.ac.uk/prospero/).

### Search methods

Database searches were conducted in May 2020 and updated in April and June 2021 to identify published studies on changes in oral microbiome during pregnancy. A medical reference librarian (DAC) developed the search strategies and retrieved citations from the following databases: Medline via PubMed, Embase via embase.com, All databases (Web of Science Core Collection, BIOSIS Citation Index, Current Contents Connect, Data Citation Index, Derwent Innovation Index, KCI-Korean Journal Database, Medline, Russian Science Citation Index, SciELO Citation Index, and Zoological Record) via Web of Science, Cochrane Central Register of Controlled Trials via Cochrane Library. A combination of text words and controlled vocabulary terms were used (oral microbiota, oral health, bacterial diversity, pregnancy, periodontal pathogens, pregnancy complication). See “ESM Appendix” for detailed search methods used.

### Inclusion and exclusion criteria

This systematic review included case–control studies, cross-sectional studies, retrospective and prospective cohort studies, randomized or non-randomized controlled trials that examined the changes of oral microorganisms in relation to pregnancy, oral diseases during pregnancy, adverse birth outcome and the effect of prenatal oral health care on oral microorganisms’ carriage. Two trained independent reviewers completed the article selection in accordance with the inclusion/exclusion criteria. Disagreements were resolved by consensus between the two reviewers or by the third reviewer.

#### Inclusion criteria

Types of participants: women during reproductive age (pregnant and non-pregnant women).

Types of intervention(s)/phenomena of interest: pregnancy.

Types of comparisons:

- oral microbiota changes throughout pregnancy;
- oral microbiota profiling between pregnancy and non-pregnancy phases;
- oral microbiota changes following prenatal oral health care;
- association between oral microorganisms during pregnancy and adverse birth outcome;
- impact of systematic or oral health conditions on oral microbiota in pregnancy.

Types of outcomes: detection and carriage of oral microorganisms, oral microbiota diversity and composition.

Types of studies: case–control study; cross-sectional study; retrospective and prospective cohort study; randomized and non-randomized controlled trials.

Types of statistical data: detection and carriage [colony forming unit (CFU)] of individual microorganisms; Confidence Intervals (CI); *p* values.

#### Exclusion criteria

In vitro studies; animal studies; papers with abstract only; literature reviews; letters to the editor; editorials; patient handouts; case report or case series, and patents.

### Data extraction

Descriptive data, including clinical and methodological factors such as country of origin, study design, clinical sample source, measurement interval, age of subjects, outcome measures, and results from statistical analysis were obtained.

### Qualitative assessment and quantitative analysis

The quality of the selected articles was assessed depending on the types of studies. For randomized controlled trials, two methodological validities were used. (1) Cochrane Collaboration’s tool for assessing risk of bias in randomized trials^31^. Articles were scaled for the following bias categories: selection bias, performance bias, detection bias, attrition bias, reporting bias, and other bias. (2) Adapted Downs and Black scoring that assesses the methodological quality of both randomized and non-randomized studies of health care interventions^32^. A total score of 26 represents the highest study quality. For cohort and cross-sectional studies, a quality assessment tool for observational cohort and cross-sectional studies was used^33^. Additionally, GRADE^34,35^ was used to assess articles used clinical interventions during pregnancy.

For the articles selected for quantitative analysis, the OpenMeta[Analyst] was used for meta-analysis (http://cebm.brown.edu/openmeta/). The 95% CI and *p* values were estimated using an unconditional generalized linear mixed effects model with continuous random effects via DerSimonian–Laird method. Heterogeneity among the studies was evaluated using *I*^2^ statistics and tested using mean difference values. Forest plots were created to summarize the meta-analysis study results of mean difference of viable counts (converted to log value) of microorganisms.

## Results

**Figure 1:**
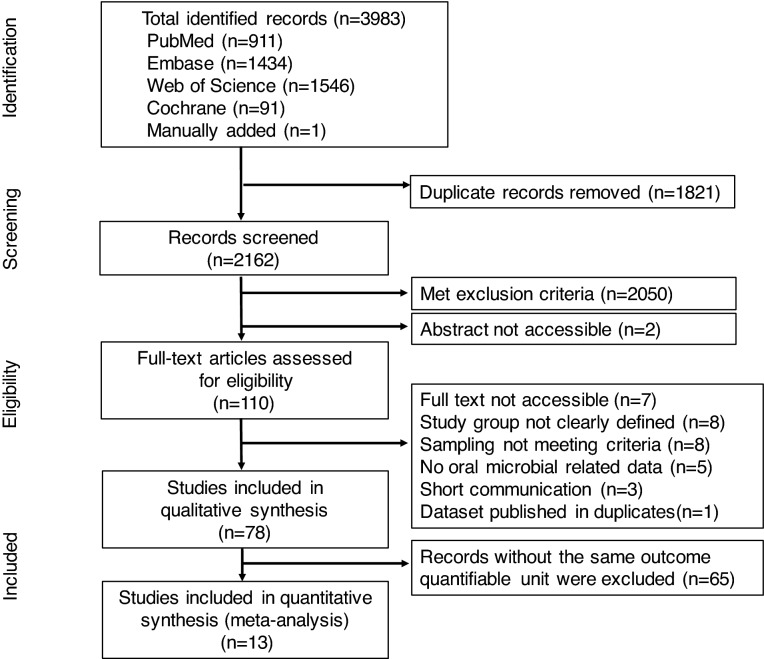
Flow diagram of study identification. The 4-phase preferred reporting items for systematic reviews and meta-analyses (PRISMA) flow diagram was used to determine the number of studies identified, screened, eligible, and included in the systematic review and meta-analysis (http://www.prisma-statement.org).

The literature analyses identified a total of 3983 records from database searches (3982) and manual additions (1). A total of 1821 duplicate references were removed. From the remaining 2162 records, 2050 were excluded after title and abstract screening. The remaining 110 studies proceeded to a full text review; 32 studies were eliminated based on the exclusion criteria and 78 articles were chosen for qualitative assessment (Fig. 1).

### Study characteristics

**Figure 2:**
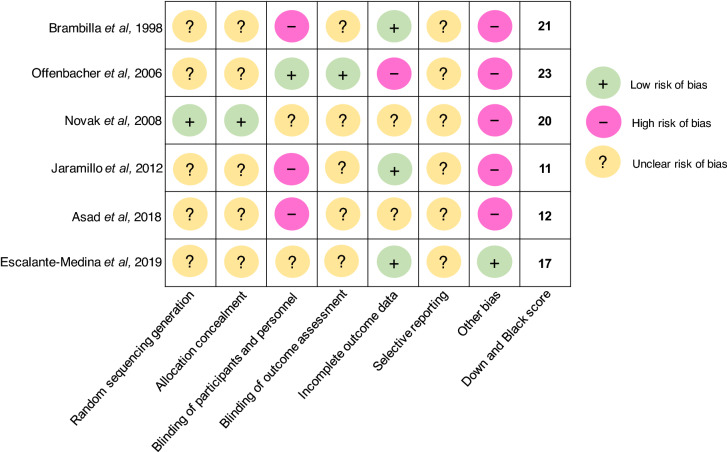
Summary of quality and risk of bias assessment using the Cochrane Collaboration’s tool for assessing risk of bias in randomized trials and the adapted Downs and Black scoring tool.

The characteristics of studies^11–17,21–25,27–29,36–98^ included in the qualitative review are summarized in Tables. A total of 78 studies are categorized into the following subgroups: 18 studies on oral microbial differences between pregnant and non-pregnant women in Table 1^14–17,36–49^; 11 studies on oral microbial differences between pregnant stages in Table 2^11–13,50–57^; 8 studies on oral microbial differences responding to prenatal dental treatment in Table 3^27–29,58–62^; 16 studies on association between oral microorganisms during pregnancy and adverse birth outcome in Table 4^21–25,63–73^; eight studies on impact of periodontal disease on oral microorganisms during pregnancy in Table 5^74–81^; six studies on impact of gestational diabetes mellitus (GDM) on oral microorganisms during pregnancy in Table 6^82–87^; 11 studies on impact of systemic health conditions on oral microorganisms during pregnancy in Table 7^88–98^. Quality and risk of bias for randomized controlled trials was assessed and are shown in Fig. 2. Quality assessment for cohort and cross-sectional studies are included in the last column of all tables.

**Table 1:** Oral microbial differences between pregnant and non-pregnant women.

Author (year)	Country, study design	Groups (no. of subjects)	Sample source	Measurement interval	Microorganisms evaluated	Microbial detection methods	Study findings	Quality assessment
Kornman and Loesche (1980)^36^	USA, prospective cohort	Pregnant (20) Non-pregnant (11)	Subgingival plaque	**Pregnant group** *T1*: < 13 weeks GA *Follow-ups*: monthly after until delivery **Non-pregnant group** Monthly visit for 4 consecutive months	*A. naeslundii, A. odontolyticus, A. viscosus, B. asaccharolyticus, P. intermedi), B. ochraceus, F. nucleatum, S. sanguis*	Culturing	The subgingival flora evolved to a composition that has more anaerobes as pregnancy progressed The anaerobe/aerobe ratio increased significantly at an early stage of pregnancy and remained high until the third trimester Only *B. melaninogenicus* ss. *intermidius* (currently *P. intermedia*) significantly increased during pregnancy compared between trimesters In the 2nd trimester, the anaerobe/aerobe ratio and the proportions of *B. melaninogenicus* ss. *intermedius* different significantly from the non-pregnant group	Fair
Muramatsu and Takaesu (1994)^37^	Japan, cross-sectional	Pregnant (19) Non-pregnant (12) Postpartum (8)	Supragingival plaque, saliva	Pregnant group One time point during pregnancy	*P. intermedia, *Black-pigmented anaerobic rods, *Actinomyces streptococcus*	Culturing	Significant differences in proportions of *Actinomyces* were found between pregnant and non-pregnant group and between 2nd trimester pregnant and postpartum group No statistically significant changes in proportions of *P. intermedia*	Fair
Yokoyama et al*.* (2008)^38^	Japan, cross-sectional	Pregnant (22) Non-pregnant (15)	Unstimulated whole saliva	**Pregnant group** 27.4 ± 5.1 weeks GA	*C. rectus, P. gingivalis, A. actinomycetemcomitans, F. nucleatum, P. intermedia*	Real-time PCR	**Positive correlations between bacteria carriage and estradiol concentrations** *C. rectus* (r = 0.443, p = 0.006) *P. gingivalis* (r = 0.468, p = 0.028) *F. nucleatum* (r = 0.452, p = 0.035) Positive correlations between *C. rectus* levels and sites of 4 mm-pocket depth (r = 0.568, p = 0.006)	Fair
Gürsoy et al*.* (2009)^16^	Finland, prospective cohort	Pregnant (30) Non-pregnant (24)	Subgingival plaque, saliva	**Pregnant group** T1: 12–14 weeks GA T2: 25–27 weeks GA T3: 34–38 weeks GA T4: 4–6 weeks postpartum; T5: After lactation **Non-pregnant group** T1–T3 (once per subsequent month)	*P. intermedia, P. nigrescens *(former *Bacteroides intermedius*)	16s rDNA sequencing and culturing	Carriage of subgingival *P. intermedia* doubled in the 2nd trimester, comparing to the 1st trimester; continued increasing till after the delivery (p < 0.05); and decreased to the lowest point after lactation Carriage of salivary *P. intermedia* remained stable during the pregnancy and decreased (p < 0.05) after lactation to the same level as the non-pregnant group *P. nigrescens* is likely associated with pregnancy gingivitis	Fair
Carrillo-de-Albornoz et al*.* (2010)^39^	Spain, prospective cohort	Pregnant (48) Non-pregnant (28)	Subgingival plaque	**Pregnant group** T1: 12–14 weeks GA T2: 23–25 weeks GA T3: 33–36 weeks GA T4: 3 months postpartum **Non-pregnant group** 2 visits 6 months apart	*C. rectus, P. gingivalis, A. actinomycetemcomitans, F. nucleatum, P. intermedia, T. forsythensis, P. micra*	Culturing	No significant changes in total bacterial counts in the pregnant group either during or after pregnancy Significant reduction in *A. actinomycetemcomitans* after delivery (p = 0.039) No statistically significant differences during pregnancy for any of the pathogens evaluated; however, significant changes from the third trimester to postpartum for all the pathogens Subjects who were positive for *P. gingivalis* had higher levels of gingival inflammation	Fair
Basavaraju et al. (2012)^40^	India, prospective cohort	Pregnant (15) Non-pregnant (15)	Subgingival plaque	**Pregnant group** T1: during pregnancy T2: 3 weeks postpartum	*Veillonella, T. forsythia, P. intermedia, P. gingivalis, Peptoscreptococcus, F. nucleatum, Propionebactierum, Mobiluncus, Candida* spp.	Culturing	The organisms which were most commonly detected in both the groups were: *Vielonella, T. forsythia, P. intermedia, P. gingivalis, Peptosreptococcus and F. nucleatum* *P. gingivalis* was present in 5 patients out of 15 in the pregnant-group as compared to 1 in the non pregnant group and the count was reduced to 3 during postpartum	Poor
Machado et al. (2012)^41^	Brazil, cross-sectional	Pregnant (20) Non-pregnant (20)	Subgingival plaque	**Pregnant group** 14–24 weeks GA	*A. actinomycetemcomitans, T. forsythia, C. rectus, P. gingivalus, T. denticola, F. nucleatum, P. intermedia, P. nigrescens*	Fluorescence in situ hybridization	No significant difference in mean total bacterial count between pregnant and non-pregnant group No significant differences between groups in the numbers of all bactieral species evaluated	Fair
Emmatty et al*.* (2013)^17^	India, cross-sectional	Pregnant (30, 10 in each trimester) Non-pregnant (10)	Subgingival plaque	**Pregnant group** One time point during pregnancy	*A. actinomycetemcomitans, P. gingivalis, P. intermedia, F. nucleatum, P. micra*	Culturing	*P. intermedia* significantly increased in pregnant women who were in their second and third trimesters as compared with first trimester and non-pregnant women Proportions of the pathogens assessed did not show any significant difference among pregnant and non-pregnant women	Fair
Borgo et al. (2014)^15^	Brazil, prospective cohort	Pregnant (9) Non-pregnant (9)	Subgingival plaque	**Pregnant group** T1: Second trimester (15–26 weeks GA) T2: Third trimester (30–36 weeks GA)	*A. actinomycetemcomitans, P. gingivalis, P. intermedia, F. nucleatum*	Real-time PCR	The detection of *A. actinomycetemcomitans* in pregnant women at 2nd and 3rd trimester was significant higher than that in the non-pregnant women (p < 0.05)	Fair
Fujiwara et al*.* (2017)^42^	Japan, prospective cohort	Pregnant (132) Non-pregnant (51)	Subgingival plaque, saliva	**Pregnant group** T1: 7–16 weeks GA T2: 17–28 weeks GA T3: 29–39 weeks GA	**Subgingival** *A. actinomycetemcomitans, P. gingivalis, P. intermedia, F. nucleatum* **Saliva** *Above 4* + *Streptococci, Staphylococci, Candida* spp.	Culturing and real-time PCR	A significant difference in total cultivable microbial number between non-pregnant and each stage of pregnancy More total bacteria counts at early stage of pregnancy (T1), comparing to the non-pregnant group (p < 0.05) Significant higher prevalence of *Candida spp.* in the middle (T2) and late (T3) pregnancy, comparing to the non-pregnant group (p < 0.05) The number of periodontal species was significantly lower in late pregnancy (T3), comparing to the early (T1) and middle (T2) pregnancy (p < 0.05) The prevalence of *P. gingivalis* and *A. actinomycetemcomitans* was significantly higher in the early (T1) and middle (T2) stage of pregnancy, comparing to the nonpregnant women (p < 0.05)	Fair
Kamate et al*.* (2017)^14^	India, prospective cohort	Pregnant (50) Non-pregnant (50)	Saliva	**Pregnant group** T1: 6 weeks GA T2: 18 weeks GA T3: 30 weeks GA T4: 6 weeks postpartum	*S. mutans*	Culturing	A significant increase in *S. mutans* during the 2nd and 3rd trimester and postpartum period of pregnancy compared to the non-pregnant group (p < 0.01)	Fair
Rio et al*.* (2017)^43^	Portugal, prospective cohort	Pregnant (30) Non-pregnant (30)	Unstimulated saliva	**Pregnant group** T1: 1st trimester T2: 3rd trimester	Yeast	Culturing	No difference in oral yeast detection within pregnancy stages and between pregnant and non-pregnant stages (p < 0.05) More oral yeast were found in the 3rd trimester than the 1st trimmest, but no difference comparing to the non-pregnant stage (p < 0.05) Saliva flow rate did not change in both groups	Fair
Lin et al*.* (2018)^44^	China, prospective cohort	Pregnant (11) Non-pregnant (7)	Supragingival plaque, saliva	**Pregnant group** T1: 11–14 weeks GA T2: 20–25 weeks GA T3: 33–37 weeks GA T4: 6 weeks postpartum **Non-pregnant group** 4 visits (same intervals of the pregnant group)	Quantity of OUT and microbiota diversity	16s rDNA sequencing	Significant higher bacterial diversity of the supragingival microbiota in third trimester compared to the non-pregnant group *Neisseriaceae* and *Porphyromonadaceae* and *Spirochaetaceae* were significantly enriched in pregnant group	Fair
Xiao et al*.* (2019)^45^	USA, cross-sectional	Low SES pregnant (48) Low SES Non-pregnant (34)	Whole non-stimulated saliva, supragingival plaque, mucosal swabs	**Pregnant group** 3rd trimester (> 28 weeks GA)	*C. albicans, C. glabrata, C. tropicalis, C. krusei, C. dubliniensis, S. mutans*	Culturing and Colony PCR	Salivary *S. mutans* carriage was higher in pregnant than non-pregnant women (p < 0.05) No difference between pregnant and non-pregnant salivary *C. albicans* carriage (p > 0.05) Tonsil (57%) was the most prevalent site for *C. albicans* detection among pregnant women Untreated decayed teeth is associated with higher carriage of salivary *S. mutans* and *C. albicans* detection in both pregnant and non-pregnant groups (p < 0.05)	Fair
Aikulola et al. (2020)^46^	Nigeria, cross-sectional	Pregnant (26) Non-pregnant (32)	Oral swab	**Pregnant group** 20–28 weeks GA	*S. aureus, N. catarrhalis, K. pneumonia, E. coli, P. melaninogenicus, P. propionicum, V. pervula, S. viridans, *Coagulase negative *Staphylococcus*	Culturing	*E. coli* was the most common species in non-pregnant group while *N. catarrhalis* was the most common in the pregnant group	Poor
Huang et al. (2020)^47^	China, cross-sectional	Pregnant (84) Postpartum (33)	Unstimulated saliva	**Pregnant group** One time point	*P. gingivalis, P. intermedia, P. nigrscens*	16s rRNA PCR	*P. nigrescens* had higher prevalence in the pregnant group (p < 0.01) *P. nigrescens* exhibited more frequently in late pregnancy than early and middle pregnancy (p < 0.05 and p < 0.01) *P. gingivalis* in the postpartum group exceeds all of the pregnant stages (p < 0.01) *P. intermedia* did not show any significant differences among groups	Fair
Sparvoli et al. (2020)^48^	Brazil, cross-sectional	Pregnant (42) Non-pregnant (18)	Oral swab	**Pregnant group** 28–36 weeks GA	Quantity of OUT and microbiota diversity	16s rRNA sequencing	Significant differences in the relative abundance of oral microbiome in pregnant women A significant dominance of *Streptococcus* and *Gemella* in pregnant women (p < 0.01 and 0 = 0.03) Shannon diversity index were higher in the non-pregnant group, while the Simpson diversity index was higher in the pregnant group	Fair
Wagle et al. (2020)^49^	Norway, cross-sectional	Pregnant (38) Non-pregnanr (50)	Saliva	**Pregnant group** 18–20 weeks GA	*S. mutans, Lactobacillus*	Culturing	*S. mutans* were more abundant in pregnant women (p = 0.03) *Lactobaciilus* did not have the significant difference between the groups	Fair

**Table 2:** Oral microbial differences between pregnancy stages.

Author (year)	Country, study design	Groups (no. of subjects)	Sample source	Measurement interval	Microorganisms evaluated	Microbial detection methods	Study findings	Quality assessment
Dasanayake et al. (2005)^50^	USA, prospective cohort	First time pregnant women (297)	Stimulated saliva	T1: 3rd trimester T2: Delivery	*S. mutans, S. sobrinus, S. sanguinus, L. acidophilus, L. casei, A. naeslundii, *Total Streptococci, Total cultivable organisms	Culturing	*A. naeslundii* gsp 2 level decreased with increased GA (p = 0.05) *L. casei* carriage increased with increased GA (p = 0.04) *L. casei* levels at the third trimester were positively associated with birth weight (β = 34.1 g; SE = 16.4; p = 0.04) Total Streptococci and total cultivable organism levels at delivery were negatively associated with birth weight After multivariate analysis with average bacterial levels, *A. naeslundii* gsp 2, *L. casei*, pregnancy age, and infant gender remained significantly associated with birth weight	Fair
Adriaens et al*.* (2009)^51^	Switzerland, prospective cohort	Healthy pregnant women (20)	Subgingival plaque	T1: 12 weeks GA T2: 28 weeks GA T3: 36 weeks GA T4: 4–6 weeks postpartum	**37 species including** *S. mutans, F. nucleatum, P. intermedia, P. gingivalis, A. actinomycetemcomitans*	DNA–DNA hybridization	*N. mucosa* increased throughout the pregnancy (p < 0.001) **Total bacterial counts** No significant differences between T1 and T2 Significant reduction from T1 to T3 (p < 0.05), and further reduction to T4 (p < 0.01) Between T1 and T4, significant differences were found for 8 of 37 species, including *S. mutans, S. aureus, polymorphum, P. micra* Between measurement intervals, no statistical differences identified for the levels of four periodontal pathogens	Fair
Molnar-Varlam et al*.* (2011)^13^	Romania, prospective cohort	Healthy pregnant women (35)	Stimulated saliva	T1: 1st trimester (11–12 weeks GA) T2: 2nd trimester (20–22 weeks GA) T3: 3rd trimester (34–35 weeks GA)	*S. mutans, Lactobacillus*	Culturing	Increase of *S. mutans* during the 2nd and 3rd trimester among women 25–35 years old Increase of *Lactobacilli* in the 2nd trimester among women 20–24 years old and 30–35 years old The salivary pH increased as the pregnancy progresses	Fair
Martinez-Pabon et al*.* (2014)^52^	Colombia, prospective cohort	Pregnant women (35)	Stimulated saliva	T1: Between 2nd and 3rd trimester T2: 7 months postpartum	*S. mutans, Lactobacillus* spp.	Culturing	No statistically significant changes in counts of *S. mutans* and *Lactobacillus* spp., but a tendency of higher numbers during pregnancy A statistically significant difference in the pH and the buffering capacity of saliva; both lower during pregnancy (p < 0.05)	Fair
DiGiulio et al*.* (2015)^11^	USA, case–control	**Pregnant women (49)** Full term (34) Preterm (15)	Saliva, vaginal, stool, oral swab from molar tooth surface & gum lines	Weekly from early pregnancy until delivery and monthly until 12 postpartum	Not specified;	16 s rDNA sequencing	The progression of pregnancy is not associated with a dramatic remodeling of the diversity and composition of a woman’s microbiota	Fair
Okoje-Adesomoju et al. (2015)^53^	Nigeria, cross-sectional	**Pregnant women (395)** 1st trimester (3) 2nd trimester (100) 3rd trimester (292)	Mucosal swab	One time point	*Klebsiella* spp., *E. coli*, *S. albus, Proteus* spp*., S. aureus, Streptococcus* spp., *Pseudomonas* spp*.*	Culturing, API 20A identification kits	*Klebsiella* species was the predominant isolate from 101 (25.6%) of the women The pattern of microbial culture whether normal for the oral cavity or not did not vary significantly with parity (p = 0.98), trimester of pregnancy (p = 0.94) or oral hygiene status (p = 0.94)	Poor
Machado et al. (2016)^54^	Brazil, prospective cohort	Healthy pregnant women (31)	Supragingival & subgingival plaque	T1: 19 ± 3.3 weeks GA; T2: 48 h postpartum; T3: 8 weeks postpartum	*T. forsythia, C. rectus, P. gingivalis, T. denticola, F. nucleatum, P. intermedia, P. nigrescens A. actinomycetemcomitans*	Fluorescence in situ hybridization	Changes in the percentage of *P. intermedia*, *F. nucleatum*, *P. gingivalis*, *T. denticola*, *C. rectus* and an increase in *A. actinomycetemcomitans* was noted, but differences were not statistically significant - A significant reduction was seen for *P. nigrescens* when all three time points were compared (p = 0.01, Friedman test), with a reduction from T1 to T3 (p = 0.002), and T2 to T3 (p = 0.037)	Fair
Balan et al. (2018)^12^	Singapore, prospective cohort	**Pregnant women (30)** 1st trimester (10) 2nd trimester (10) 3rd trimester (10)	Subgingival plaque, unstimulated saliva	T1: 1st trimester (< 12 weeks GA) T2: 2nd trimester (21–24 weeks GA) T3: 3rd trimester (32–36 weeks GA) T4: 6 weeks postpartum	12 Phyla, 65 genera, 131 species	16s rDNA sequencing	Species richness and diversity of the subgingival plaque and saliva samples were relatively stable across the pregnancy The abundance of *Prevotella, Streptococcus* and *Veillonella* in both subgingival plaque and saliva samples were more during pregnancy A significant decline in the abundance of pathogenic species, e.g., *Veillonella parvula*, *Prevotella species* and *Actinobaculum species,* was observed from pregnancy to postpartum period	Fair
Goltsman et al*.* (2018)^55^	USA, retrospective cohort	**Pregnant (10)** Term delivery (6) Preterm (4)	Saliva, vaginal, stool, rectal swabs	Every 3 weeks over the course of gestation	1553 taxa	16 s rDNA sequencing	Alpha diversity, both inter-individual and intra-individual, remained stable across the pregnancy and postpartum	Fair
de Souza Massoni et al. (2019)^56^	Brazil, cross-sectional	**Pregnant (52)** 1st trimester (16) 2nd trimester (21) 3rd trimester (15) Non-pregnant (15)	Subgingival plaque	One time point	*A. actinomycetemcomitans, P. gingivalis, T. forsythia, S. oralis, *Universal	qPCR	No significant differences in total amount of bacteria between the groups *T. forsythia* showed significant differences in quantification between 1st trimester and 3rd trimester, and 1st trimester and non-pregnant (p = 0.048 and p = 0.014) Amount of *T. forsythia* positively correlated with the diagnosis of gingivitis in pregnant women (p = 0.031)	Fair
Dunlop et al*.* (2019)^57^	USA, retrospective cohort	African American Pregnant women (122) Oral samples (97)	Vaginal, oral (tongue, hard palate, gum line) and rectal swabs	T1: 8–14 weeks GA T2: 24–30 weeks GA	Not specified	16S rDNA sequencing	No difference in Chao1 and Shannon diversity for the vaginal, oral, or gut microbiome across pregnancy for the group overall For the oral microbiota, having a low level of education and receipt of antibiotics between study visits were associated with greater Bray–Curtis dissimilarity, with some attenuation of the effect of education when additionally controlling for prenatal antibiotics	Fair

**Table 3:** Oral microbial differences responding to prenatal dental treatment.

Author (year)	Country, study design	Groups (no. of subjects)	Sample source	Measurement interval	Microorganisms evaluated	Microbial detection methods	Study findings	Quality assessment
Brambillia et al*.* (1998)	Italy, RCT	**Treatment group (33)** Dietary counseling + Dental Prophy + systematic fluoride (1 mg per day from the last week of 6th month GA) + daily fluoride and CHX mouth rinse **Control group (32)** Dietary counseling + Dental Prophy + systematic fluoride (1 mg per day from the last week of 6th month GA)	Unstimulated saliva	T1: 3rd month GA T2: 6th month GA T3: 9th month GA T4: 6 months postpartum T5-T7: 12, 18, 24 months postpartum, respectively	*S. mutans*	Culturing	A reduction in salivary *S. mutans* levels in treatment group became significant (p < 0.01) six months after the study began (at T3); *S. mutans* reduction remained significant (p < 0.001) at the end of the study Children of mothers in treatment group had significantly lower salivary *S. mutans* levels than those of control-group mothers at 18 months old (p < 0.05) and 24 months old (p < 0.01)	See Fig. 2
Mitchell-Lewis et al*.* (2001)^59^	USA, prospective cohort	**Treatment group (74)** Prenatal Periodontal intervention (Hygiene instruction + full mouth debridement) **Control group (90)** Postpartum periodontal intervention	Subgingival plaque	**Treatment group** T1: During pregnancy **Control group** T1: After delivery	*P. gingivalis, P. intermedia, P. nigrescens, B. forsythus, A. actinomycetemcomitans, F. nucleatum, T. denticola, P. micros, C. rectus, E. corrodens, E. nodatum, S. intermedius*	DNA-DNA hybridization checkerboard method	Mothers who had pre‐term low birth weight had significantly higher levels of *B. forsythus* and *C. rectus*, and elevated counts for the other species examined	Fair
Offenbacher et al. (2006)^60^	USA, RCT	**Treatment group (40)** SRP + polishing + OHI + sonic power toothbrush *during 2nd trimester* **Control group (34)** (Supragingival debridement + manual toothbrush during pregnancy) + (SRP 6 weeks postpartum)	Gingival cervical fluid, subgingival plaque	T1: < 22 weeks GA T2: Postpartum	***Red cluster*** *P. gingivalis, T. forsythensis, T. denticola* **Orange cluster** *F. nucleatum, P. intermedia, P. nigrescens, C. rectus, A. actinomycetemcomitans*	DNA-DNA hybridization checkerboard method	No significant changes from baseline to postpartum in the levels of any single bacterial species or cluster among control mothers *P. intermedia* and *P. nigrescens* reduction detected in the treatment group (p < 0.05) A composite score of orange-cluster organisms decreased in treatment group (p = 0.03)	See Fig. 2
Novak et al*.* (2008)^61^	USA, RCT	**Treatment group (413):** SRP before 21 weeks GA **Control group (410):** SRP after delivery	Subgingival plaque	T1: 13–16 weeks GA T2: 29–32 weeks GA	*P. gingivalis, T. denticola, T. forsythia, P. intermedia, C. rectus, F. nucleatum, A. actinomycetemcomitans*	Realtime PCR	Women in treatment group had significantly greater reductions (p < 0.01) in counts of *P. gingivalis*, *T. denticola*, *T. forsythia*, *P. intermedia*, and *C. rectus* than untreated women	See Fig. 2
Volpato et al*.* (2011)^27^	Brazil, prospective cohort	**Treatment group (30)** Oral Environment Stabilization (atraumatic caries excavation and fillings + extraction of retained roots)	Saliva	T1: Before treatment (70% in 2nd trimester) T2: 1 week after treatment	*S. mutans*	Culturing	A statistically significant decrease (p < 0.0001) in *S. mutans* counts between saliva samples before and after oral environment stabilization	Fair
Jaramillo et al. (2012)^29^	Colombia RCT	**Pregnant women with preeclampsia (57)** Treatment group (26): SRP Control group (31): Supragingival prophy	Subgingival fluid	T1: Before treatment T2: Postpartum	*P. gingivalis, P. intermedia, P. nigrescens, T. forsythia, C. rectus, E. Corrodens, D. pneumosinte*s, *A. actinomycetemcomitans*	PCR	The detection of assessed microorganisms did not decrease following periodontal treatment in control group and intervention group	See Fig. 2
Asad et al*.* (2018)^28^	Pakistan, RCT	Pregnant women with a minimal of 3 decayed teeth **Treatment group (32):** atraumatic restorative treatment **Control group (32):** no treatment	Stimulated saliva	T1: Before treatment T2: 1 week after treatment	*S. mutans*	Realtime PCR	Salivary *S. mutans* was reduced after the atraumatic restorative treatment (p < 0.001) Salivary *S. mutans* remained the same level between the two study time point in the control group (p = 0.29)	See Fig. 2
Escalante-Medina et al*.* (2019)^62^	Peru, RCT	**Treatment group (23):** toothpaste with 10% xylitol **Control group (22):** toothpaste without xylitol	Saliva	T1: Before the use of xylitol toothpaste T2: 14 days after the use of the toothpaste	*S. mutans*	Culturing	No difference in *S. mutans* among the pregnant women who used xylitol toothpaste compared to those who used toothpaste without xylitol (p = 0.062) Both toothpastes, with and without xylitol, were effective to decrease the count of *S. mutans* in the saliva of pregnant women (p = 0.001 and p = 0.005, respectively)	See Fig. 2

**Table 4:** Association between oral microorganisms during pregnancy and adverse birth outcome—preterm delivery.

Author (year)	Country, study design	Groups (no. of subjects)	Sample source	Measurement interval	Microorganisms evaluated	Microbial detection methods	Study findings	Quality assessment
Hasegawa et al*.* (2003)^63^	Japan, cross-sectional	**Pregnant women (88)** Threatened premature labor Full term (22) Preterm (18) Healthy (48)	Subgingival plaque	Not specified	*A. actinomycetemcomitans, P. gingivalis, P. intermedia, T. forsythia*	PCR	-Detection of *T. forsythia was significantly higher among* Threatened premature labor preterm delivery group than the full-term group (p < 0.05)	Fair
Dörtbudak et al. (2005)^21^	Austria, cross-sectional	**Women at risk for miscarriage or preterm delivery (36)** Preterm delivery (6) Full-term delivery (30)	Amniotic fluid, vaginal smears and dental plaque	15–20 weeks GA	***Red cluster*** *P. gingivalis, T. forsythensis, T. denticola* **Orange cluster:** *F. nucleatum, P. intermedia, P. nigrescens, C. rectus*	Culturing, PCR	Detection of pathogens in orange and red clusters of subgingival plaque samples was lower in full-term group (16.7%) compared to preterm group (83.3%) (p < 0.01) Carriage of pathogens orange and red clusters of subgingival plaque samples was higher in preterm group (p < 0.01) The levels of Amniotic IL-6 and PGE2 were significantly higher in women delivering pre-term (p < 0.001); Amniotic IL-6 (r = 0.56, p < 0.01) and PGE2 (r = 0.50, p < 0.01) cytokine levels were correlated with subgingival bacterial counts	Poor
Lin et al. (2007)^64^	USA, nested case–control	**Women with periodontal disease (31)** Preterm delivery (14) Full-term delivery (17)	Subgingival plaque	T1: 22 weeks GA T2: Postpartum	*P. gingivalis, P. intermedia, P. nigrescens, T. forsythensis, T. denticola, C. rectus,F. nucleatum, A. actinomycetemcomitans*	Checkerboard DNA–DNA hybridization	Postpartum bacterial carriage difference between preterm and full-term groups * P. gingivalis*, *T. forsythensis*, *P. intermedia*, and *P. nigrescens* (p < 0.05) * T. denticola* and *C. rectus* (p < 0.065) Patients with a high level of *C. rectus* at T1 showed a non-significant tendency to have a higher risk for preterm births (odds ratio [OR] = 4.6; 95% confidence interval [CI] 0.99–21.1)	Fair
Durand et al. (2009)^65^	USA, case–control	**Pregnant women (107)** Preterm delivery (34) Full-term delivery (73)	Saliva	One time point at recruitment (from 1st trimester to 8 weeks postpartum)	*S. mutans*, *Lactobacilli* spp.	Culturing using commercially kit (CRT bacteria®)	Preterm group had lower level of *Lactobacilli* (p = 0.009) No difference in *S. mutans* carriage between preterm and full-term groups (p = 0.053)	Fair
Hasegawa et al*.* (2011)^66^	Japan, cross-sectional	**High risk (hospitalized) Pregnant women (23)** Normal birth weight (8) Low birth weight (15)	Saliva and Subgingival plaque	2nd trimester	*P. gingivalis*	PCR	*P. gingivalis* was detected in saliva among 7 out the 15 low birth weight group, and 3 of the 8 normal delivery group *P. gingivalis* was detected in plaque among 8 out the 15 low birth weight group, and 4 of the 8 normal delivery group No report on statistical data regarding oral *P. gingivalis* and birth weight	Fair
Sadeghi et al. (2011)^67^	Iran. prospective cohort	**Pregnant women (243)** Premature delivery (10) Full-term delivery (233)	Saliva	20–30 weeks GA	*Gram-positive and negative cocci, Gram-positive and negative bacilli, Spirilla, Spirochetes, Fusiform bacteria, Actinomycetes, Yeasts*	Culturing, Bacteria gram staining	A significant statistical difference between the mean of gram-negative cocci and intrauterine fetal death cases (p = 0.04) A significant relationship in the presence of spirochetes in saliva between premature and normal delivery (p < 0.05) No significant relationship for other bacteria	Fair
Cassini et al*.* (2013)^22^	Italy, prospective cohort	**Pregnant women (80)** Preterm delivery (8) Full-term delivery (72)	Subgingival plaque, vaginal samples	14–30 weeks GA (One time point for microbial analysis)	*A. actinomycetemcomitans, P. gingivalis, T. forsythia, T. denticola,* *F. nucleatum, P. intermedia*	Realtime PCR	The amount of subgingival *P. gingivalis* of preterm women was higher than that of term women None of assessed periodontopathogen resulted as correlated to preterm low birthweight	Fair
Ye et al. (2013)^23^	Japan, cross-sectional	**Pregnant women (95)** *Threatened premature labor (TPL)* Preterm delivery (13) Full-term delivery (34) *Healthy women* Preterm delivery (1) Full-term delivery (47)	Subgingival plaque, unstimulated saliva and peripheral blood	26–28 weeks GA	*A. actinomycetemcomitans, P. gingivalis*, *T. denticola*	ELISA	*P. gingivalis* detection was more frequently detected among preterm group than full-term group among TPL women No significant difference in detection frequency of *A. actinomycetemcomitans*, *P. gingivalis* and *T. denticola* between TPL and healthy groups	Good
Andonova et al*.* (2015)^24^	Croatia, case–control	**Pregnant women (70)** Preterm delivery (30) Full-term delivery (40)	Subgingival plaque	28–36 + 6 weeks GA	*P. gingivalis, P. intermedia, F. nucleatum, Bacteroides* sp., *Veillonela* sp., *P. micros, S. intermedius, A. actinomycetemcomitans E. lentum*	Culturing	A sevenfold higher risk of development of preterm delivery in women with periodontal anaerobes in subgingival plaque than women without Levels of *P. gingivalis, F. nucleatum, A. actinomycetemcomitans* were statistically significantly higher in preterm births compared to full-term deliveries	Fair
Hassan et al*.* (2016)^68^	Saudi Arabia, Prospective cohort	**Pregnant women (94)** Preterm delivery (22) Full-term delivery (72)	Subgingival plaque	2nd trimester	*P. oralis, V. parvula, P. melanionogenica, P. anaerobius, P. asaccharolticus, C. subterminate, C. perfringens, C. clostridioforme, C. bifermentans, E. lenta, A. meyeri*	Culturing	*A. meyeri* and *C. bifermentans* were significantly associated with higher odds of preterm birth (11.2 and 5.1), with the estimate of *C. bifermentans* showing greater precision (95% confidence interval = 1.5, 17.5) (p < 0.05)	Fair
Usin et al*.* (2016)^69^	Argentina, cross-sectional	**Pregnant women (134)** Preterm low birth weight delivery (18) Full-term normal birth weight delivery (116)	Subgingival plaque	3rd trimester	*P. gingivalis, P. intermedia, T. forsythia, T. denticola, A. actinomycetemcomitans*	PCR	*P. gingivalis* and *T. denticola* were significantly more prevalent in Full-term normal birth weight delivery group	Fair
Costa et al*.* (2019)^25^	Brazil, case–control	**Pregnant women (330)** Preterm delivery (110) Full-term delivery (220)	Gingival crevicular fluid, blood	T1: During pregnancy T2: at the time of delivery	*P. gingivalis, P. intermedia, F. nucleatum, A. actinomycetemcomitans*	DNA-DNA hybridization	Higher periodontopathogenic bacteria burden (PBB) did not increase the risk of preterm birth	Fair
Gomez et al*.* (2020)^70^	Colombia, case–control	**Pregnant women (94)** Adverse birth outcome (23) Non-adverse birth outcome (17)	Subgingival plaque, placental samples	During pregnancy	*P. gingivalis, T. forsythia, T. denticola, E. nodatum, A. actinomycetemcomitans, F. nucleatum*	PCR	*P. gingivalis*-related placenta infection with adverse pregnancy outcome group reflects high levels of IFN-γ with a significative decreasing of NK-related cytokines (p < 0.05)	Good
Ye et al*.* (2020)^71^	Japan, prospective cohort	**Pregnant women (64)** Threatened preterm labor (TPL) (Low birth weight) (9) Threatened preterm labor (Normal weight delivery) (19) Control (36)	Saliva, Subgingival plaque, placental samples	During pregnancy	*P. gingivalis, P. intermedia, T. forsythia, T. denticola, A. actinomycetemcomitans, F. nucleatum*	qPCR, ELISA	Quantity of *P. gingivalis* and *T. forsythia* in plaque samples and detection frequency of *P. intermedia* in saliva were higher in TPL- Low birthweight delivery than those in TPL-Healthy delivery group and/or in control-healthy delivery group	Good
Ye et al*.* (2020)^72^	Japan, prospective cohort	**Pregnant women (95)** Threatened preterm labor (TPL) (Low birthweight) (14) Threatened preterm labor (Healthy delivery) (33) Control (48)	Saliva, Subgingival plaque, placental samples	26–28 weeks GA	*P. gingivalis*	qPCR	The detection frequency of *P. gingivalis* in plaque and placenta were significantly correlated with low birthweight delivery in TPL group. In the receiver operating characteristic curve analysis, an amount of *P. gingivalis* in plaque ≥ 86.45 copies showed a sensitivity of 0.786 and a specificity of 0.727 (AUC 0.792) for predicting low birthweight delivery in TPL	Good
Ye et al*.* (2020)^73^	China, prospective cohort	**Pregnant women (90)** Preterm low birth weight (PLBW) (22) Healthy delivery (68)	Saliva	2nd trimester	*P. gingivalis, P. intermedia, T. forsythia, T. denticola, A. actinomycetemcomitans, F. nucleatum, E. saphenum, Fretibacterium* sp., *R. dentocariosa* *Human oral taxon (HOT) 360, TM7 sp. HOT 356*	Culturing, qPCR, ELISA	There was no significant difference in periodontal parameters and serum IgG levels for periodontal pathogens between PLBW and healthy delivery (HD) groups The amount of *E. saphenum* in saliva and serum IgG against *A. actinomycetemcomitans* were negatively correlated with PLBW	Good

**Table 5:** Impact of periodontal disease on oral microorganisms during pregnancy.

Author (year)	Country, study design	Groups (no. of subjects)	Sample source	Measurement interval	Microorganisms evaluated	Microbial detection methods	Study findings	Quality assessment
León et al. (2007)^74^	Chile, cross-sectional	**Women with threatened premature labor (26)** Gingivitis (8) Periodontitis (12) No-periodontal disease (6)	Amniotic fluid and subgingival plaque	24–34 weeks GA	*A. actinomycetemcomitans, P. gingivalis, P. intermedia, P. nigrescens, E. corrodens, F. nucleatum, Capnocytophaga species, C. rectus, M. micros*	Culturing, PCR	Subgingival plaque samples including *P. gingivalis* were found in 50.0% (13/26) of patients No difference for *P. gingivalis* detection between groups with or without periodontal diseases	Fair
Santa Cruz et al. (2013)^75^	Spain, prospective cohort	**Pregnant women (170)** Periodontitis (54) Non-periodontitis (116)	Subgingival plaque	8–26 weeks GA	*A. actinomycetemcomitans, P. gingivalis, P. intermedia, P. nigrescens, T. forsythia, P. micra, C. rectus, F. nucleatum, E. corrodens, Capnocytophaga* spp.	Culturing	Periodontitis was associated with higher detection of *F. nucleatum* (97.4%), *P. intermedia* & *P. nigrescens* (94.9%), *P. gingivalis* (76.9%) and *P. micra* (56.4%) with high proportions of microbiota for *P. gingivalis* (18.9%), *P. intermedia* & *P. nigrescens* (3.9%) or *F. nucleatum* (5.5%)	Fair
Tellapragada et al., (2014)^76^	India, cross-sectional	**Pregnant women (390)** Gingivitis (147) Periodontitis (40) No-periodontal disease (203)	Subgingival plaque	8–24 weeks GA	*P. gingivalis*, *P. intermedius*, *P. nigrescens*, *T. forsythia*, *A. actinomycetemcomitans*, *C. rectus*, *C. ochracea*, *C. sputigens*, *E. corrodens*, *T. denticola*	PCR	Women with periodontitis had a higher detection of *P. gingivalis*, *P. intermedius*, *P. nigrescens*, *T. denticola* (p < 0.05)	Fair
Lima et al*.* (2015)^77^	Brazil, cross-sectional	**Pregnant women (86)** Periodontitis (9) Gingivitis (27) Non-periodontitis (50)	Gingival crevice sample	During pregnancy	*P. gingivalis, T. forsythia, T. denticola, P. intermedia*	PCR	Socransky Red Complex (*P. gingivalis*, *T. forsythia and T. denticola)* was not present in pregnant women with healthy periodontium Socransky Red Complex was present in pregnant women with gingivitis (3.7%) and in a higher percentage of pregnant women with periodontitis (33.3%)	Fair
Lu et al*.* (2016)^78^	China, cross-sectional	**Pregnant women (72)** Periodontitis (36) Non-periodontitis (36)	Saliva	During pregnancy	*P. gingivalis*, *A. actinomycetemcomitans, F. nucleatum*, *P. intermedia*, *T. forsythia*, *T. denticola, *Epstein–Barr virus, Cytomegalovirus, herpes simplex virus	PCR	The detection rates of included periodontopathic microorganisms were not significantly different between the two groups (p > 0.05) The coinfection rate of EBV and *P. gingivalis* was significantly higher in the case group than in the control group (p = 0.028)	Good
Yang et al. (2019)^79^	USA, cross-sectional	**Pregnant women (34)** Gingivitis (12) Non-gingivitis (22)	Saliva and subgingival plaque	3rd trimester	Multiple taxa	16S rDNA sequencing and qPCR	No significant differences in alpha diversity (Chao1 or Shannon index) between groups (p > 0.05) *Prevotella* and *Leptotrichia* were more prevalent in healthy participants, whereas *Mogibacteriaceae*, *Veionella* and *Prevotella* were more prevalent in participants in the gingivitis group (p < 0.01)	Fair
Balan et al*.* (2020)^80^	China, cross-sectional	**Pregnant women (20)** Gingivitis (10) Non gingivitis (10) **Non-pregnant women (10)**	Subgingival plaque	21–24 weeks GA	Multiple taxa	16S rDNA sequencing and qPCR	In term of alpha and beta diversity, minimal differences were observed between pregnant women with and without gingivitis Oral bacterial community showed higher abundance of pathogenic taxa during healthy pregnancy as compared with nonpregnant women despite similar gingival and plaque index scores	Fair
Tanneeru et al. (2020)^81^	India, cross-sectional	**Pregnant women with preeclampsia (200)** With periodontal disease (100) Without periodontal disease (100)	Subgingival plaque, placental samples	During pregnancy	*P. gingivalis, F. nucleatum, P. intermedia, T. forsythia, T. denticola, *Epstein–Barr virus, Cytomegalovirus, herpes simplex virus	PCR	*T. forsythia*, *T. denticola*, *F. nucleatum*, and EBV were detected more in the groups with periodontal diseases in their subgingival samples	Poor

**Table 6:** Impact of gestational diabetes mellitus (GDM) on oral microorganisms during pregnancy.

Author (year)	Country, study design	Groups (no. of subjects)	Sample source	Measurement interval	Microorganisms evaluated	Microbial detection methods	Study findings	Quality assessment
Dasanayake et al*.* (2008)^82^	USA, Nested case–control	**Predominately Hispanic Pregnant women (262)** With GDM (22) Without GDM (240)	Subgingival plaque, blood, cervico-vaginal samples	18.2–3.4 weeks GA	*C. rectus, F. nucleatum* ssp., *Nucleatum, T. forsythia, P. gingivalis, T. denticola*	PCR	The level of evaluated microorganisms from subgingival plaque had no difference between GDM and non-GDM groups (p > 0.05)	Fair
Ganiger et al*.* (2019)^83^	India, case–control	**Pregnant women (60)** With GDM (124) Without GDM (325)	Subgingival plaque	During pregnancy	*P. gingivalis, P. intermedia*	PCR	*P. gingivalis* were more frequently detected among women with GDM group (80%) than those ones without GDM (40%) (p < 0.05)	Fair
Yao et al. (2019)^84^	China, case–control	**Pregnant women (449)** With GDM (124) Without GDM (325)	Supragingival and subgingival plaque	14–28 weeks GA	*Streptococci, Lactobacilli, Tuberculosis bacilli*, black-pigmented bacteria, *Capnocytophagia*, *Actinomycetes*, *E. coli, S. aureus, P. aeruginosa K. pneumoniae, A. actinomycetemcomitans, C. albicans*	Culturing	No detection difference between GDM and non-GND groups: *streptococci, lactobacilli, actinomycetes*, *E. coli*, *S. aureus* and *P. aeruginosa* (p > 0.05) Higher detection in GDM group: *Tuberculosis bacilli* (p = 0.000), Black-pigmented bacteria (p = 0.026), and *Capnocytophaga* (p = 0.030) The total number of oral anaerobic bacteria (p = 0.000), tuberculosis bacilli (p = 0.000), Black-pigmented bacteria (p = 0.007), *Capnocytophaga* (p = 0.000), and *Actinomycetes* (p = 0.000) was more among GDM group	Fair
Crusell et al*.* (2020)^85^	Denmark, prospective cohort	**Pregnant women (211)** With GDM (50) Without GDM (161)	Unstimulated saliva	T1: 27–33 weeks GA T2: 9 months postpartum	Multiple taxa	16S rDNA sequencing	Shannon’s diversity and Pielou’s evenness decreased from pregnancy to postpartum, regardless of GDM status (p = 0.0008, p = 0.001, p = 0.007 respectively) During pregnancy (T1), no difference in richness, overall diversity or evenness between GDM and non-GDM women	Fair
Xu et al*.* (2020)^96^	China, Case–control	**Pregnant women (60)** With GDM (30) Without GDM (30)	Saliva and fecal sample	3rd trimester	Multiple taxa	16S rDNA sequencing	The GDM cases showed lower α-diversity, increased *Selenomonas* and *Bifidobacterium*, an decreased *Fusobacteria* and *Leptotrichia* in oral microbiota	Fair
Li et al*.* (2021)^87^	China, case–control	**Pregnant women (111)** With GDM (42) Without GDM (69)	Saliva and plaque	3rd trimester	Multiple taxa	16S rDNA sequencing	Certain bacteria (e.g. combination of *Lautropia* and *Neisseria* in dental plaque and *Streptococcus* in saliva) in either saliva or dental plaque can effectively distinguish women with GDM from healthy pregnant women	Good

**Table 7:** Impact of systemic health conditions on oral microorganisms during pregnancy.

Author (year)	Country, study design	Groups (no. of subjects)	Sample source	Measurement interval	Microorganisms evaluated	Microbial detection methods	Study findings	Quality assessment
Contreras et al*.* (2006)^88^	Colombia, case–control	**Pregnant women (373)** Pre-eclampsia (130) Non-pre-eclampsia (243)	Subgingival plaque	26–36 weeks GA	*A. actinomycetemcomitans, P. gingivalis, P. intermedia, P. nigrescens, T. forsythia, Campylobacter* spp.,* Eubacterium *spp.,* Fusobacterium *spp.,* P. micros, E. corrodens, D. pneumosintes, b-hemolyticstreptococci, Staphylococci *spp., yeast	Culturing	The prevalence of *P. gingivalis, T. forsythensis*, and *E. corrodens* was higher in the preeclampsia group (61.5%, 28.5%, and 49.2%, respectively) than the non-preeclampsia group (p < 0.01) Periodontal disease and chronic periodontitis were more prevalent in the pre-eclampsia group (p < 0.001)	Fair
Herrera et al. (2007)^89^	Columbia, case–control	**Pregnant women (398)** Pre-eclampsia (145) Non-pre-eclampsia (253)	Subgingival plaque	28–36 weeks GA	*A. actinomycetemcomitans, P. gingivalis, P. intermedia, P. nigrescens, T. forsythia, Campylobacter *spp.,* Eubacterium *spp.,* Fusobacterium *spp.,* P. micros, E. corrodens, D. pneumosintes, b-hemolyticstreptococci, Staphylococci *spp., yeasts	Culturing	*P. gingivalis* and *E. corrodens* were more prevalent in the pre-eclamptic women than in healthy group (p < 0.001) All other species studied had non-statistically significant differences between pre-eclamptic group and healthy controls	Fair
Ávila et al*.* (2011)^90^	Brazil, cross-sectional	**Pregnant women (140)** Rheumatic valve disease (70) Healthy (70)	Saliva	2nd–3rd trimester	*A. actinomycetemcomitans, P. gingivalis, T. forsythia*	PCR	The proportion of *P. gingivalis* was significantly higher in the saliva of healthy pregnant women (p = 0.004), but not in other species	Fair
Merglova et al*.* (2012)^91^	Czech Republic, Case–control	**Pregnant women (142)** High risk pregnancy (81) Healthy (61)	Stimulated saliva	3rd trimester	*S. mutans*	Culturing	High levels of *S. mutans* in the saliva in over 70% of subjects in high-risk pregnancy group	Poor
Stadelmann et al. (2015)^92^	Switzerland, prospective case–control	**Pregnant women (56)** Premature Rupture of Membranes (PPROM) (32) Healthy (24)	Gingival crevicular fluid, subgingival plaque and vaginal samples	T1: 20–35 weeks GA T2: 48 h postpartum T3: 4–6 weeks postpartum	*A. actinomycetemcomitans, P. gingivalis, T. forsythia, T. denticola, P. intermedia, P. micra, F. nucleatum, F. necrophorum, C. rectus, E. nodatum, E. corrodens, Capnocytophaga* species	MicroIDent®plus11 test (PCR, reverse hybridization)	In PPROM group, there was a statistically significant decrease from T1 to T2 for the microbiological group of major periodontopathogens (*A. actinomycetemcomitans*, *P. gingivalis*, *T. denticola*, *T.* forsythia; p = 0.0313) and also for the group of all analyzed bacteria (p = 0.0039) There were no statistically significant differences between groups at any timepoint (p > 0.05) The prevalence of grouped subgingival periodontopathogenic bacteria did not change overtime in the control group (p > 0.05)	Fair
Paropkari et al*.* (2016)^93^	USA, cross-sectional	**Pregnant women (22)** Smoker (11) Non-smoker (11) **Non-pregnant women (22)** Smoker (11) Non-smoker (11)	Subgingival plaque	21–24 weeks GA	Multiple taxa	16S-pyrotag sequencing	Alpha diversity (Shannon index) was not significantly different between all groups (p > 0.05) Pregnant smokers demonstrated clusters that were not seen in either pregnant women or in smokers, e.g., *Bradyrhizobium *spp*., Herbaspirillum, E. coli, Prevotella melalinogenica*, *Prevotella *spp*.,* *Corynebacterium* spp*., Dialister *spp*. * *Tannerella *spp*.* Species belonging to the genera *Pseudomonas, Acidovorax, Enterobacter, Enterococcus, Diaphorobacterium*, *Methylobacterium* demonstrated significantly greater abundances in pregnant women (both smokers and nonsmokers)	Fair
Jaiman et al*.* (2018)^94^	India case–control	**Pregnant women (30)** Pre-eclampsia (15) Non-pre-eclampsia (15)	Subgingival plaque and placental blood	During pregnancy	*P. gingivalis, F. nucleatum*	Culturing	No statistically significant association between microorganism in plaque and placental blood between normotensive control and preeclamptic pregnant women	Poor
Parthiban et al*.* (2018)^95^	India case–control	**Pregnant women (50)** Pre-eclampsia (25) Non-pre-eclampsia (25)	Subgingival plaque and placental samples	During pregnancy	*A. actinomycetemcomitans, P. gingivalis, T. forsythia, P. intermedia*	qPCR	The subgingival plaque samples of pre-eclamptic women showed significantly higher frequencies of *P. intermedia*	Fair
Tuominen et al. (2018)^96^	Finland, case–control	**Pregnant women (40)** HPV positive (20) HPV negative (20)	Mucosal scrapings of oral cavity, and cervix, placenta	3rd trimester	Multiple taxa	PCR and 16S rDNA sequencing	Species with increased relative abundance in HPV positive oral samples: *Selenomonas* spp. (p = 0.0032), *Megasphaera* spp. (p = 0.026) and *TM73* (p = 0.018) Species with decreased relative abundance in HPV positive oral samples: *Haemophilus* spp. (p = 0.019) HPV positive oral samples displayed higher richness (Chao1 index) (p = 0.0319), but no difference in diversity (Shannon index), comparing to HPV negative samples	Fair
Tanneeru et al*.* (2020)^97^	India, cross-sectional	**Pregnant women (200)** Pre-eclampsia with periodontitis (100) Pre-eclampsia without periodontitis (100)	Subgingival plaque and placental samples	During pregnancy	*P. gingivalis, F. nucleatum, P. intermedia, T. forsythia, T. denticola*	PCR	Association between periodontal bacteria (*P. gingivalis*, *F. nucleatum*, *P. intermedia*, *T. forsythia*) and preeclampsia (detailed data not shown in the article)	Poor
Wang et al*.* (2020)^98^	China, cross-sectional	**Pregnant women (61)** Hypothyroidism (30) Healthy (31)	Saliva and fecal samples	During pregnancy	Multiple taxa	16S rDNA sequencing	The oral cavity of pregnant women in the hypothyroidism group had higher relative abundances of *Gammaproteobacteria, Prevotella, Neisseria*, and *Pasteurellaceae*, whereas that of women in the control group had higher relative abundances of *Firmicutes, Leptotrichiace,* and *Actinobacteria*	Fair

*GA* gestational age, *SES* social economic status, *RCT* randomized controlled trial, *SRP* scaling and root planning.

The quality of the selected articles was assessed using two methodological validities: (1) Cochrane Collaboration’s tool for assessing risk of bias in randomized trials^31^. (2) Adapted Down and Black scoring^32^ that assess the methodological quality of both randomized and non-randomized studies of health care interventions. A total score of 26 represents the highest study quality.

### Oral microbial differences between pregnant and non-pregnant women

Evident changes of oral microbiota were seen among pregnant women, comparing to those of non-pregnant women. A significantly higher amount of total cultivable microorganisms were found in pregnant women comparing to the non-pregnant at each stage of pregnancy^42^. The plaque bacterial community was more diverse in 3rd trimester pregnant women compared to non-pregnant women^44^.

Regarding oral pathogens, the prevalence of *A. actinomycetemcomitans* was significantly higher in pregnant women in each stage compared to non-pregnant women (*p* < 0.05)^15,42^. Two studies^14,45^ assessed *S. mutans* carriage in saliva, and found that *S. mutans* carriage increased significantly throughout the pregnancy; particularly, significant differences were seen between women in their first trimester and non-pregnant women (*p* < 0.01^14^ and *p* < 0.05^45^). The detection of *P. gingivalis* and *P. intermedia* increased significantly in pregnant women compared to non-pregnant women^17,42^. Although no difference was found in terms of *C. albicans* carriage between pregnant and non-pregnant women^45^, two studies revealed a higher detection of *Candida* spp. among women in their late pregnancy stage, comparing to the non-pregnant group^42,43^.

### Oral microbial differences throughout pregnancy stages

Interestingly, seven studies^11,12,51,52,54,55,57^ revealed a stable oral microbial community during pregnancy. All four studies^11,12,55,57^ that performed sequencing analysis revealed that microbiota species richness, diversity and composition were relatively stable across the pregnancy stages. The level of *S. mutans* and *Lactobacillus* spp. were assessed in two studies^13,52^. The levels of *S. mutans* and *Lactobacilli* increased in both studies, but without statistical signficance^52^.

Some studies^12,39,51^ indicated significant differences from pregnancy to the postpartum period. A total bacterial count reduced significantly after delivery (*p* < 0.01)^51^. Several species, like *S. mutans* and *Parvimonas micra*, showed significant differences in postpartum compared to the early stages of pregnancy^51^. This finding was also noticed in another study where *A. actinomycetemcomitans*, *P. gingivalis*, *Tannerella forsythia*, *P. micra* showed an abrupt decline after delivery^39^. *A. actonomycetemcomitans*, especially, dropped significantly in its amount after delivery (*p* = 0.039)^39^. A significant decline in the abundance of pathogenic species from pregnancy to postpartum period was observed as well^12^.

### Impact of prenatal dental treatment on maternal oral flora

Four studies^27,28,58,62^ revealed lower *S. mutans* carriage in the group with oral health care intervention during pregnancy compared to the control group. Fluoride and chlorhexidine treatment as a caries-preventive regimen during pregnancy showed a statistical difference in the salivary *S. mutans* levels between the study and control groups by the end of the 3-month treatment period^58^. At the end of the pregnancy, the reduction in *S. mutans* level was still significant in the study group (*p* < 0.01)^58^.

Two studies^27,28^ which conducted oral environmental stabilization, including atraumatic restorative treatment, revealed statistically significant decrease in *S. mutans* (*p* < 0.0001^27^ and *p* < 0.001^28^) before and after the intervention. Comparatively, there was no significant reduction in salivary *S. mutans* count in the group who did not get the treatment (*p* = 0.29)^28^. Interestingly, children of treated group mothers had significantly lower salivary *S. mutans* levels than those of untreated group mothers (*p* < 0.05)^58^.

Periodontal pathogenic microbiomes did not reveal consistent results. Three studies^29,60,61^ performed SRP as treatment. Some microbiomes had significantly greater reductions where counts of *P. gingivalis*, *P. intermedia*, *T. denticola*, *T. forsythia*, and *C. rectus* was significantly lower in treated women (*p* < 0.01)^61^. A similar result was also found with detection of *P. intermedia* and *P. nigrescens* reduced significantly in the treatment group (*p* < 0.05)^60^. Yet, the study by Jaramillo et al.^29^ did not detect a significant decrease in the levels of bacterial species between treated and untreated groups. Quality of evidence and strength of recommendation by GRADE assessment is described in ESM Appendix 4. Quality of evidence was assessed with the study design and factors to either increase or reduce the quality for clinical interventional studies. Strength of recommendation was evaluated based on whether all individuals will be best served by the recommended course of action. Depending on whether the course is conditional or discretionary, the recommendation was given either strong or weak.

### Impact of periodontal disease on oral microorganisms during pregnancy

Three studies^75,79,80^ did not identify any significant findings that the clinical periodontal condition and the levels of subgingival microbiome during pregnancy are related to pregnancy complications.

However, when subgingival plaque in women with threatened premature labor was assessed, *P. gingivalis* was found in the half of patients with periodontal disease^74^. The presence of *Eikenella corrodens* and *Capnocytophaga* spp. were significantly related to preterm birth and low birth weight respectively (*p* = 0.022 and *p* = 0.008)^75^. No statistical significance was found in overall microbiome diversity in comparison of healthy gingiva and gingivitis groups. However, bacterial taxa like *Mogibacteriaceae* and genera *Veillonella* and *Prevotella* were more prevalent in the gingivitis group^79^.

### Association between oral microorganism during pregnancy and adverse birth outcome

Five studies^22–24,71,72^ showed that the amount of *P. gingivalis* in subgingival plaque was significantly higher in women with preterm birth than women with term birth. Also, CFU counts of red and orange complex pathogens, in which *P. gingivalis* belongs, from dental plaque in women with preterm delivery was significantly higher (*p* < 0.01)^21^. The levels of *Fusobacterium nucleatum*, *T. forsythia*, *Treponema denticola*, and *A. actinomycetemcomitans* were highly related to the preterm births compared to term deliveries^22,24^.

However, higher periodontopathogenic bacteria burden did not increase the risk of preterm birth, despite the increase in periodontal disease activity^25^. The levels of microorganisms like *P. gingivalis*, *T. forsythensis*, *T. denticola*, *P. intermedia*, and *F. nucleatum* were not significantly higher in the preterm group than in the term group^64^.

### Impact of systemic diseases on oral microorganism during pregnancy

#### Gestational diabetes mellitus (GDM)

Two studies^82,85^ did not find significant differences in either clinical periodontal disease nor in the diversity and richness between women with GDM and non-GDM. The detection rate and the number of oral bacteria in women with GDM were higher than in non-GDM women, especially in the second trimester of pregnancy^84^. Oral bacterial detection rate and total number in several species, such as black-pigmented bacteria*,* were significantly higher in pregnant women with GDM than those in non-diabetic pregnant women^84^. Conversely, oral bacterial detection of oral *streptococci* and *lactobacilli* did not show any significant differences^84^.

#### Pre-eclampsia

Two studies^88,89^ performed in Colombia and three studies^81,94,95^ performed in India revealed the influence of pre-eclampsia on the levels of the oral microbiome. Specifically, the birth weight of newborns were significantly lower in women with pre-eclampsia (*p* < 0.001)^88^. *P. gingivalis* and *E. corrodens* were more prevalent in the pre-eclampsia group than in the control group^88,89^. Further, the women with pre-eclampsia had a higher frequency of periodontal disease and chronic periodontitis (*p* < 0.001)^88^.

#### Preterm premature rupture of membranes (PPROM)

No statistically significant differences in the oral microbiome were observed in women with PPROM and those without at any time of measurement. However, in the PPROM group, significant decreases in the level of major periodontopathogens were noted from 20 to 35 weeks of gestation to within 48 h after parturition^92^.

#### Rheumatic valvular disease, smoking, and HPV

The frequency of periodontal disease in women with rheumatic valvular disease was not significantly different compared to women without the disease^90^. Smoking was associated with lower levels of gram negative facultative and higher levels of gram-negative anaerobes^93^. The presence of HPV infection and potential pathogens in oral microbiota composition were positively associated^96^.

### Meta-analysis

A limited number of studies were included for meta-analysis due to the requirement of the same comparisons and outcome measures. Meta-analyses were performed to assess differences of total bacteria carriage, periodontal or cariogenic pathogens between pregnant and non-pregnant women, or between pregnancy stages, and following prenatal dental treatment.

**Figure 3:**
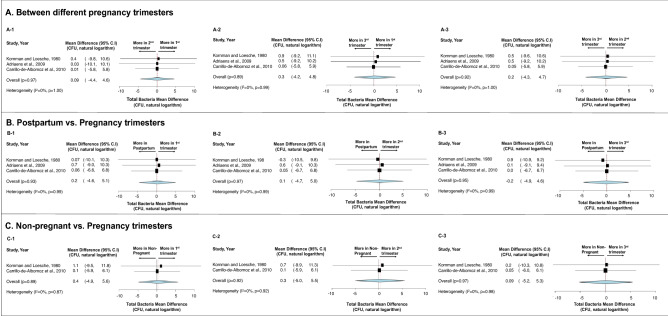
Impact of pregnancy status on subgingival plaque total bacterial carriage. (**A**) Mean difference of total bacterial carriage in subgingival plaque between different trimesters of pregnancy. (**B**) Mean difference of total bacterial carriage in subgingival plaque between pregnancy and postpartum. (**C**) Mean difference of total bacterial carriage in subgingival plaque between pregnant women and non-pregnant women. Study heterogeneity (I^2^) and the related *p* value were calculated using the continuous random effect methods. The Mean Difference, 95% CI of each study included in the meta-analyses and forest plots of comparisons shown in A-1 through C-3 indicate that, regarding total bacterial carriage in subgingival plaque, there is no statistically difference between each stage of pregnancy (p > 0.05), between postpartum and pregnancy (p > 0.05), and between non-pregnant and pregnant women (p > 0.05).

**Figure 4:**
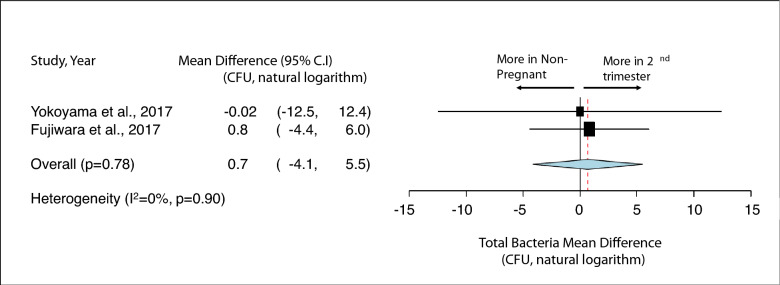
Impact of pregnancy status on salivary total bacterial carriage. Mean Difference of salivary total bacterial carriage in non-pregnant and 2nd trimester pregnant women. Study heterogeneity (I^2^) and the related *p* value were calculated using the continuous random effect methods. The Mean Difference, 95% CI of each study included in the meta-analysis and forest plot of comparisons indicate that, regarding salivary total bacterial carriage, there is no statistically significant difference between non-pregnant and 2nd trimester pregnant women (*p* > 0.05).

**Figure 5:**
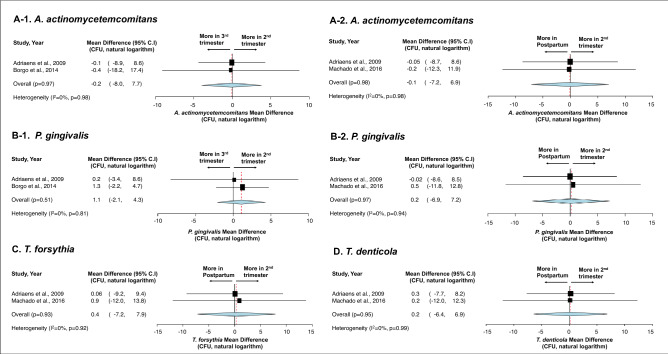
Impact of pregnancy status on the carriage of periodontal pathogens in subgingival plaques. (**A**) Carriage of *A. actinomycetemcomitans* during pregnancy trimesters (**A-1**) and between pregnancy and postpartum (**A-2**). (**B**) Carriage of *P. gingivalis* during pregnancy trimesters (**B-1**) and between pregnancy and postpartum (**B-2**). (**C**) Carriage of *T. forsythia* between postpartum and 2nd trimester. (**D**) Carriage of *T. denticola* between postpartum and 2nd trimester. Study heterogeneity (I^2^) and the related *p* value were calculated using the continuous random effect methods. The Mean Difference, 95% CI of each study included in the meta-analyses and forest plots of comparisons shown in (**A**–**D**) indicate that, regarding the carriage [measured by colony forming unit (CFU)] of four different periodontal pathogens in subgingival plaque, there is no statistically significant difference between stages of pregnancy and between postpartum and pregnancy (p > 0.05).

**Figure 6:**
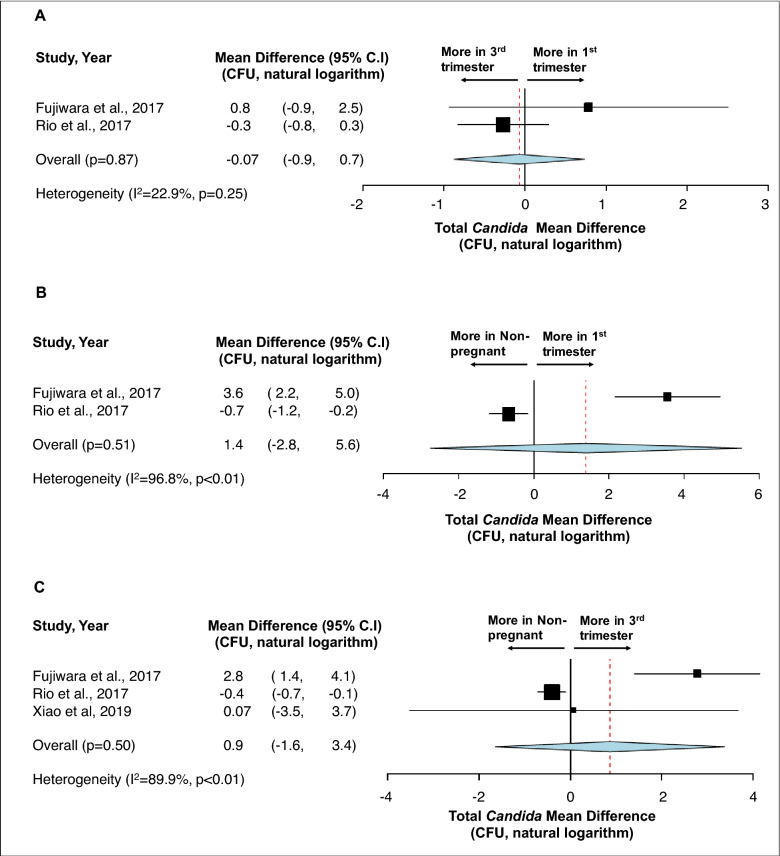
Impact of pregnancy status on salivary *Candida* carriage. The Mean differences of *Candida* carriage between 1st and 3rd trimester (**A**), between non-pregnancy and 1st trimester (**B**), and between non-pregnancy and 3rd trimester (**C**) indicated that oral *Candida* remain stable during the pregnancy and no differences (p > 0.05) are detected between pregnant and non-pregnant women. Study heterogeneity (I^2^) and the related *p* value were calculated using the continuous random effect methods.

**Figure 7:**
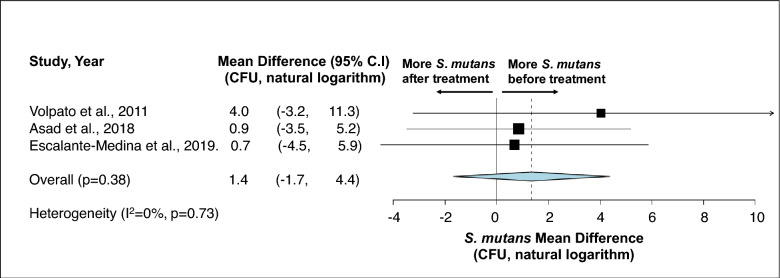
Effect of prenatal dental treatment on salivary *S. mutans* reduction. A meta-analysis was performed on two studies that assessed salivary *S. mutans* carriage before and after receiving prenatal dental treatment. Study heterogeneity (I^2^) and the related *p* value were calculated using the continuous random effect methods. The Mean Difference, 95% CI of each study included in the meta-analysis and forest plot of comparison indicate that, regarding salivary *S. mutans* carriage, there is no statistically significant difference before and after prenatal dental treatment (p = 0.38).

First, no statistical difference was detected in terms of total bacteria carriage in subgingival plaque (Fig. 3)^36,39,51^ and saliva (Fig. 4)^38,42^ between different stages of pregnancy and between pregnant and non-pregnancy groups. Second, although more subgingival periodontal pathogens (*P. gingivalis*, *T. forsythia*, and *T. denticola*) were seen among pregnant women in their early stage of pregnancy, and more *A. actinomyctemcomitans* was seen in the later stage of pregnancy and in postpartum, no statistical significance was detected between groups (Fig. 5)^15,51,54^. Third, regarding oral *Candida*, no statistical difference was seen throughout the pregnancy and between non-pregnant and pregnant women (Fig. 6)^42,43,45^. Lastly, the effects of prenatal dental treatment on salivary *S. mutans* carriage were evaluated in three studies (Fig. 7)^27,28,62^. Although no significant difference was found, the reduction of salivary *S. mutans* was reported upon receiving prenatal dental treatment.

## Discussion

### Are pregnant women at more risk for oral disease due to oral microbial changes?

Our study examined the currently available literature that reported oral microbial changes in relation to pregnancy. A fair number of studies reported an increased carriage of total oral bacteria and some disease-specific oral pathogens among pregnant women compared to the non-pregnant or postpartum group. However, meta-analyses only confirmed an increased total bacterium in saliva among pregnant women. Undetected statistical differences of subgingival total bacteria counts and specific oral pathogens between comparing groups could be due to a limited data set. Future studies are warranted to obtain conclusive findings of the association between pregnancy and oral microbial changes.

The oral cavity represents a substantial and diversified microbiota as a result of various ecologic determinants^9^. The cluster of oral microorganisms harmonizes to maintain oral microbial balance through a symbiotic relationship with their host in a state of health^9,99^. This balance has a crucial role in maintaining functions and fighting against infections in the oral cavity^99^. An imbalanced oral microbial community environment could lead to overgrowth of pathogenic bacteria or opportunistic pathogens, causing oral diseases, such as dental caries and periodontal diseases^7,8^. Previous studies suggested that during pregnancy, women are at higher risk for oral diseases^14^, due to the hormonal changes, such as estrogen, progesterone, relaxin, and gonadotropin^100^, and the increased pH in oral cavity from vomiting and craving snacks with high sugar^28^. It is speculated that pregnancy presents as a special physiological state for women, which could induce changes of the normal flora in the oral cavity^1,2^. For instance, the significantly higher detection of *P. gingivalis* and *P. intermedia* during pregnancy explains the tendency of more significant gingival inflammation in pregnant women^15,44^. Furthermore, the elevation of *A. actonomycetemcomitans* and *P. gingivalis* during the early stage of pregnancy predispose pregnant women to be at higher risk for periodontal diseases^42^.

### Are oral microorganisms harbinger for adverse birth outcome?

Our study also evaluated the association between adverse birth outcomes and the oral microbial community. A significant question is whether oral microbial changes in pregnancy could be a harbinger for adverse birth outcomes. High levels of periodontal pathogens during pregnancy were evidently associated with an increased risk for preterm delivery^24,64^. The level of *P. gingivalis*, specifically, was higher in the preterm delivery group in three studies^22–24^. This bacterium could potentially influence a diagnosis of threatened premature labor through invasion of the amniotic cavity due to the presence in both the subgingival and respective amniotic fluid samples in those pregnant women with an increased risk^74^. Women with pre-eclampsia who developed an adverse birth outcome tended to have more diagnoses of periodontal disease with higher *P. gingivalis* and *E. corrodens*^88^. Hence, careful monitoring of expectant mothers with pre-eclampsia is advised to prevent further complications related to birth outcomes. However, a lack of meta-analysis due to insufficient consistent data suggests that further studies are needed to clarify the role of the microbial change in pregnancy as related to adverse birth outcome.

Preterm birth is defined as the birth of a baby before 37 weeks gestational age^22,23^. Many identified risk factors for low birth weight and preterm birth have been identified, such as maternal age, hypertension, usage of drug, alcohol or tobacco, genetics or environmental factors^101^. Also, early studies stated that periodontal inflammation is associated with pregnancy complications by affecting systemic inflammation from anaerobes and gram-negative periodontopathic bacteria^20,63,102^. More recent studies, however, reported no association with increased risk of adverse birth outcomes with periodontal bacteria^103,104^. As much as this topic is controversial, included studies described different results as well. Some studies showed that women with preterm delivery had a higher level of few microorganisms^21–24,74^; whereas alternatively, other studies did not succeed to present a positive relationship between higher subgingival bacterial level and the risk of adverse birth outcome^25,64,75,79^.

Interestingly, a few studies revealed that preterm birth prevalence was lower among women who had dental cleaning during pregnancy and that periodontal treatment provided to mothers with mild to moderate periodontal disease before 21 gestational weeks may reduce preterm births by 6%^105,106^. Considering these results, some may quickly conclude that these treatments are effective and have benefits in lowering adverse birth outcomes. However, it is still inconclusive how these procedures bring changes in the microbiological levels.

### How systemic and oral diseases during pregnancy impact oral flora?

GDM is diabetes or any degree of glucose intolerance occurring during pregnancy^84^, and one of the most common obstetric complications, seen in 7% of all pregnancies in the United States every year^82^. GDM is associated with adverse birth outcomes and long-term consequences for pregnant women and their child^85^. The increased risk of future metabolic disorders in women with GDM has been studied^85^. Also, recent reports indicated that hyperglycemic pregnant women have an altered placental microbiota compared with normoglycemic pregnant women^107,108^. Consequently, risk of disorders in the offspring may be increased with changed salivary microbiota influenced by GDM, which affects the placental microbiota. Pregnant women with GDM should be carefully monitored for periodontal diseases^84^, since both diseases are associated with adverse birth outcomes^109,110^. However, the positive correlation between GDM and the altered oral microbial community is unclear.

Therefore, further studies on this topic are highly encouraged to provide sufficient quantitative data to predict the power and demonstrate this relationship at a demographic level since particular ethnic communities, such as Native Americans, Asians, and Hispanics, present higher prevalence than African Americans and Caucasians^85^.

### Does prenatal dental treatment lead to modified oral microflora?

Routine dental care during pregnancy has been recommended as important and safe to perform by multiple medical and dental professional organizations^111,112^. Prenatal dental treatment includes dental prophylaxis, dental fillings to restore decayed teeth, root canal therapy and extractions for severely decayed and/or periodontally compromised teeth^1^. Maintaining good prenatal oral health is essential for mothers and their offspring^1^, since maternal oral health is strongly associated with children’s oral health. However, due to various barriers, such as lack of awareness, social hardships, lack of access to prenatal care, prenatal dental care is largely underutilized. Xiao et al*.* reported that more than 80% of underserved US pregnant women have at least one untreated decayed tooth, and average number of decayed teeth is 3.9^45^. Similar data indicates that more than 70% of underserved pregnant women in Florida have unmet oral health needs^113^.

Despite the importance of prenatal dental care to the mothers and their children, the magnitude of benefits in obtaining prenatal oral health care, particularly, the modification of oral flora towards a healthier composition, has not been classified. Although the majority of studies indicated a lower carriage of *S. mutans* after receiving oral health care intervention and prevention^27,28,58^, the result from the meta-analysis does not indicate statistically significant changes of *S. mutans* following prenatal dental treatment. The fact that only two studies^27,28^ were included in the meta-analysis should be taken into consideration. Interestingly, studies^29,60,61^ that provided SRP to pregnant women had inconsistent results with the changes in the detection of periodontal pathogens. However, different microbial detection methods, measurement interval, subject groups should be considered.

Nonetheless, despite a wide range of prenatal dental treatment provided, ranging from fluoridation to oral environment stabilization, pregnant women in most of these reported studies did achieve oral disease-free status before delivery. Future clinical studies and clinical trials that provide total oral rehabilitation during pregnancy are warranted to comprehensively assess prenatal dental care's impact on maternal oral flora. Positive results will provide more evidence to support providing prenatal oral health care to mothers, which may potentially lead to a reduction in the vertical transmission of cariogenic bacteria and fungi to children^58^.

## Limitations

The following limitations should be cautiously considered when interpreting the results of this review: (1) studies included utilized inconsistent and heterogeneous approaches in grouping study data and reporting findings. Various methodologies for detecting and analyzing microorganisms were reported. The dissimilarity of recording the carriage of microorganisms, e.g., total counts, detection rate in percentages of different species of bacteria, frequency, normalization of the CFU data by using log_10_ (CFU/mg), for example, complicates the comparison of findings and data across the studies. Therefore, conducting a meta-analysis for each subgroup becomes unlikely, and this compromises a better quantitative understanding of the data; (2) variability of methodologies for bacteria and yeast quantification. As the quantification of bacteria and yeast was the meta-analysis outcome measure in this systematic review, it is worth noting that clinical sample collection and processing methods can significantly affect these microbiological outcomes. In addition, since both culture-dependent and culture-independent methods were used to detect and quantify multiple microorganisms, different levels of sensitivity and specificity across the studies are seen and reflected in the heterogeneity of studies included in the meta-analysis. Standardized methods for both identification and quantification are needed to ensure comparable results while enhancing study reproducibility; (3) due to the lack of study subject’s data on other possible determinants, e.g., race, ethnicity, demographic, socioeconomic, etc., the meta-analyses performed in this review did not adjust potential confounders mentioned above when comparing mean difference in CFUs, which might under- or over-estimate the effect of pregnancy on oral microflora; (4) as most of the studies did not report sample size calculation, study power to detect differences is questionable.

## Conclusions

In summary, studies have shown that the oral microflora during pregnancy stages remain relatively stable; however, distinctive patterns of microorganisms’ presence and abundance have been observed between pregnancy and postpartum stages and between pregnant and non-pregnant women. Oral microflora during pregnancy appears to be influenced by oral and systemic disease status. Given prenatal dental care decreases specific oral pathogens, more studies are needed to define the outcome magnitude. Future efforts are needed to understand pregnancy and its relationship with the oral microbial community and the association between maternal oral microflora and adverse birth outcomes. Gaining knowledge on this topic could contribute to modifying health care strategies and policies at both community and individual levels to improve mother and child health outcomes.

## Supplementary Information

Supplementary Information.
