# Supplementary material for: Oral microflora and pregnancy: a systematic review and meta-analysis
Source: Sci Rep. 2021 Aug 19;11:16870. doi: 10.1038/s41598-021-96495-1 (PMC8377136; doi:10.1038/s41598-021-96495-1)
Supplement: Supplementary file 1 — Supplementary Information. [file 41598_2021_96495_MOESM1_ESM.pdf]

Oral Microflora during Pregnancy and Its Association with Health Conditions: A Systematic Review and Meta-Analysis

Hoonji Jang[1], Alexa Patoine[1], Tong Tong Wu[2], Daniel A. Castillo[3], Jin Xiao[1*]

[1] Eastman Institute for Oral Health, University of Rochester Medical Center, Rochester, NY, USA

[2] Department of Biostatistics and computational biology, University of Rochester Medical Center, Rochester, USA

[3] Miner Library, University of Rochester Medical Center, Rochester, NY, USA

***Corresponding author:** Jin Xiao

Jin Xiao DDS, MS, PhD
Associate Professor
Director, Perinatal Oral Health
Eastman Institute for Oral Health
University of Rochester
625 Elmwood Ave, Rochester, USA,14620
Email: jin_xiao@urmc.rochester.edu
Phone: +1 585-273-1957

Appendix 1: Search methods

PubMed Strategy

(("Mouth"[Mesh] OR "Saliva"[Mesh] OR "Mouth Diseases"[Mesh] OR "Tooth Diseases"[Mesh] OR "Gingival Crevicular Fluid"[Mesh] OR "Periodontal Index"[Mesh] OR "Mouth"[tiab] OR "Mouths"[tiab] OR "Dental"[tiab] OR "Teeth"[tiab] OR "Tooth"[tiab] OR "Periodont*"[tiab] OR "Gingiv*"[tiab] OR "Subgingiv*"[tiab] OR "Supragingiv*"[tiab] OR "Gum"[tiab] OR "Gums"[tiab] OR "Caries"[tiab] OR "Saliv*"[tiab] OR "Decayed, Missing, and Filled Teeth"[tiab] OR "DMFT"[tiab]) AND (("Microbiota"[Mesh] OR "Porphyromonas Gingivalis"[Mesh] OR "Streptococcus Mutans"[Mesh] OR "Streptococcus"[Mesh] OR "Candida Albicans"[Mesh] OR "Microbiot*"[tiab] OR "Micro-biot*"[tiab] OR "Biota"[tiab] OR "Microbiom*"[tiab] OR "Metagenomic*"[tiab] OR "Microflor*"[tiab] OR "Flora"[tiab] OR "Biofilm*"[tiab] OR "Bio-film*"[tiab] OR "CFU"[tiab] OR "Colony-forming Unit*"[tiab] OR "Dysbio*" [tiab] OR "Periodontopathogen*"[tiab] OR "Periodontal pathogen*"[tiab]) OR "Porphyromonas Gingivalis"[tiab] OR "P. Gingivalis"[tiab] OR "Streptococcus Mutans"[tiab] OR "S. Mutans"[tiab] OR "Candida Albicans"[tiab] OR "C. Albicans"[tiab] OR (("Ecologic*" [tiab] OR "Bacter*"[tiab] OR "Microb*"[tiab] OR "Pathogen*"[tiab] OR "Colon*"[tiab]) AND ("Shift*"[tiab] OR "Load*"[tiab] OR "Prevalenc*"[tiab] OR "Count*"[tiab] OR "Number*"[tiab] or "Amount*"[tiab] OR "Level*"[tiab] OR "Sampl*"[tiab] OR "Swab*"[tiab])))) AND ("Pregnancy"[Mesh] OR "Pregnant Women"[Mesh] OR "Pregnancy Trimesters"[Mesh] OR "Prenatal Care"[Mesh] OR "Pregnancy Outcome"[Mesh] OR "Pregnancy Complications"[Mesh] OR "Obstetric Labor Complications"[Mesh] OR "Premature Birth"[Mesh] OR "Pre-Eclampsia"[Mesh] OR "Pregnan*"[tiab] OR "Prenatal Care"[tiab] OR "Childbear*"[tiab] OR "Child Bear*"[tiab] OR "Gestat*"[tiab] OR "Gravidit*"[tiab] OR "Prenat*"[tiab] OR "Pre-nat*"[tiab] OR "Antenat*"[tiab] OR "Ante-nat*"[tiab] OR "Fetal"[tiab] OR "Foetal"[tiab] OR "Foetus*"[tiab] OR "Fetus*"[tiab] OR "Antepart*"[tiab] OR "Intrauterine Death*"[tiab] OR "Intrauterine Demise"[tiab] OR "Stillbirth*"[tiab] OR "Still birth*"[tiab] OR "Spontaneous Abortion*"[tiab] OR "Tubal Abortion*"[tiab] OR "Early Pregnancy Loss*"[tiab] OR "Miscarriage*"[tiab] OR "Pregnancy Outcome*"[tiab] OR "Birth Outcome*"[tiab] OR "Labor Outcome*"[tiab] OR "Labour Outcome*"[tiab] OR "Obstetric*"[tiab] OR "Pre Eclamp*"[tiab] OR "Preeclamp*"[tiab] OR "Preclamp*"[tiab] OR "Edema Proteinuria Hypertension"[tiab] OR "EPH"[tiab] OR "PPROM"[tiab] OR (("Prematur*"[tiab] OR "Pre matur*"[tiab]) AND ("Labor*"[tiab] OR "Labour*"[tiab] OR "Deliver*"[tiab] OR "Birth*"[tiab] OR "Childbirth*"[tiab] OR ("Ruptur*"[tiab] AND "Membran*"[tiab])))) NOT ("Animals"[Mesh] NOT ("Animals"[Mesh] AND "Humans"[Mesh])) NOT ("Review" [Publication Type] OR "Letter" [Publication Type] OR "Editorial" [Publication Type] OR "Case Reports" [Publication Type])

Embase Strategy

('mouth'/exp OR 'saliva'/exp OR 'mouth disease'/exp OR 'periodontal index'/exp OR 'dmft index'/exp OR (mouth* OR dental OR teeth OR tooth OR periodont* OR gingiv* OR subgingiv* OR supragingiv* OR gum OR gums OR caries OR saliv* OR decayed-missing-or-filled-teeth OR dmft):ti,ab) AND ('microflora'/exp OR 'porphyromonas gingivalis'/exp OR 'streptococcus mutans'/exp OR 'candida albicans'/exp OR (microbiot* OR micro-biot* OR biota OR microbiom* OR metagenomic* OR microflor* OR flora OR biofilm* OR bio-film* OR cfu OR colony-forming unit* OR dysbio* OR periodontopathogen* OR periodontal-pathogen* OR porphyromonas-gingivalis OR p.-gingivalis OR streptococcus-mutans OR s.-mutans OR candida-albicans OR c.-albicans OR ((ecologic* OR bacter* OR microb* OR pathogen* OR colon*) NEAR/3 (shift* OR load* OR prevalenc* OR count* OR

number* OR amount* OR level* OR sampl* OR swab*))):ti,ab) AND ('pregnancy'/exp OR 'pregnant woman'/exp OR 'prenatal care'/exp OR 'pregnancy outcome'/exp OR 'pregnancy complication'/exp OR 'labor complication'/exp OR 'prematurity'/exp OR 'preeclampsia'/exp OR (pregnan* OR prenatal-care OR childbear* OR child-bear* OR gestat* OR gravidit* OR prenat* OR pre-nat* OR antenat* OR ante-nat* OR fetal OR foetal OR foetus* OR fetus* OR antepart* OR intrauterine-death* OR intrauterine-demise OR stillbirth* OR still-birth* OR spontaneous-abortion* OR tubal-abortion* OR early-pregnancy-loss* OR miscarriage* OR pregnancy-outcome* OR birth-outcome* OR labor-outcome* OR labour-outcome* OR obstetric* OR pre-eclamp* OR preeclamp* OR preclamp* OR edema-proteinuria-hypertension OR eph OR pprom OR (ruptur* NEAR/3 membran*) OR ((prematur* OR pre-matur*) NEAR/3 (labor* OR labour* OR deliver* OR birth* OR childbirth*))):ti,ab) NOT ([animals]/lim NOT [humans]/lim) AND ('article'/it OR 'article in press'/it OR 'chapter'/it OR 'conference paper'/it OR 'erratum'/it OR 'short survey'/it)

Web of Science Strategy
TS=(mouth OR saliva OR mouth-disease OR periodontal-index OR dmft-index OR (mouth* OR dental OR teeth OR tooth OR periodont* OR gingiv* OR subgingiv* OR supragingiv* OR gum OR gums OR caries OR saliv* OR decayed OR missing OR filled OR teeth OR dmft)) AND TS=(microflora OR porphyromonas-gingivalis OR streptococcus-mutans OR candida-albicans OR (microbiot* OR micro-biot* OR biota OR microbiom* OR metagenomic* OR microflor* OR flora OR biofilm* OR bio-film* OR cfu OR colony-forming-unit* OR dysbio* OR periodontopathogen* OR periodontal-pathogen* OR porphyromonas-gingivalis OR p.-gingivalis OR streptococcus-mutans OR s.-mutans OR candida-albicans OR c.-albicans OR ((ecologic* OR bacter* OR microb* OR pathogen* OR colon*) NEAR/2 (shift* OR load* OR prevalenc* OR count* OR number* OR amount* OR level* OR sampl* OR swab*)))) AND TS=(pregnancy OR pregnant woman OR prenatal care OR pregnancy outcome OR pregnancy complication OR labor complication OR prematurity OR preeclampsia OR pregnan* OR prenatal-care OR childbear* OR child-bear* OR gestat* OR gravidit* OR prenat* OR pre-nat* OR antenat* OR ante-nat* OR fetal OR foetal OR foetus* OR fetus* OR antepart* OR intrauterine-death* OR intrauterine-demise OR stillbirth* OR still-birth* OR spontaneous-abortion* OR tubal-abortion* OR early-pregnancy-loss* OR miscarriage* OR pregnancy-outcome* OR birth-outcome* OR labor-outcome* OR labour-outcome* OR obstetric* OR pre-eclamp* OR preeclamp* OR preclamp* OR edema-proteinuria-hypertension OR eph OR pprom OR (ruptur* NEAR/2 membran*) OR ((prematur* OR pre-matur*) NEAR/2 (labor* OR labour* OR deliver* OR birth* OR childbirth*))) Refined by: [excluding] DOCUMENT TYPES: (PATENT OR EDITORIAL MATERIAL OR REVIEW OR CASE REPORT OR ABSTRACT OR LETTER OR MEETING)

Cochrane Database Strategy
("Mouth" OR "Mouths" OR "Dental" OR "Teeth" OR "Tooth" OR "Periodont*" OR "Gingiv*" OR "Subgingiv*" OR "Supragingiv*" OR "Gum" OR "Gums" OR "Caries" OR "Saliv*" OR "Decayed, Missing, and Filled Teeth" OR "DMFT") AND (("Microbiot*" OR "Micro-biot*" OR "Biota" OR "Microbiom*" OR "Metagenomic*" OR "Microflor*" OR "Flora" OR "Biofilm*" OR "Bio-film*" OR "CFU" OR "Colony-forming Unit*" OR "Dysbio*" OR "Periodontopathogen*" OR "Periodontal pathogen*") OR "Porphyromonas Gingivalis" OR "P. Gingivalis" OR "Streptococcus Mutans" OR "S. Mutans" OR "Candida Albicans" OR "C. Albicans" OR (("Ecologic*" OR "Bacter*" OR "Microb*" OR "Pathogen*" OR "Colon*") NEAR/3 ("Shift*" OR "Load*" OR "Prevalenc*" OR "Count*" OR "Number*" or "Amount*" OR "Level*" OR "Sampl*" OR "Swab*")))) AND ("Pregnan*" OR "Prenatal Care"[Mesh] OR "Childbear*" OR "Child Bear*" OR "Gestat*" OR "Gravidit*" OR "Prenat*" OR "Pre-

nat*" OR "Antenat*" OR "Ante-nat*" OR "Fetal" OR "Foetal" OR "Foetus*" OR "Fetus*" OR "Antepart*" OR "Intrauterine Death*" OR "Intrauterine Demise" OR "Stillbirth*" OR "Still birth*" OR "Spontaneous Abortion*" OR "Tubal Abortion*" OR "Early Pregnancy Loss*" OR "Miscarriage*" OR "Pregnancy Outcome*" OR "Birth Outcome*" OR "Labor Outcome*" OR "Labour Outcome*" OR "Obstetric*" OR "Pre Eclamp*" OR "Preeclamp*" OR "Preclamp*" OR "Edema Proteinuria Hypertension" OR "EPH" OR "PPROM" OR ("Ruptur*" AND "Membran*") OR (("Prematur*" OR "Pre matur*") NEAR/3 ("Labor*" OR "Labour*" OR "Deliver*" OR "Birth*" OR "Childbirth*")))

Appendix 2: Data extraction form

Reviewer: ____________________________Date: __________________________________
Study ID: ____
Title: ____
Lead author (First and Corresponding): ____
Publication year: ______________Country:_________________Language:___________
Journal: ____
Aim of the study: ____

Part I: Study Methods

Design:

- ☐ Randomized clinical trial
- ☐ Non-randomized clinical trial
- ☐ other (describe)____________________

Origin of the study:
City_______________ State/Province________________ Country:________________

Subject recruitment site:

- ☐ Hospital/Dental School clinic
- ☐ Community
- ☐ Epidemiology Study site
- ☐ Other (describe) ___________________________

Study visits: ___________________ ☐ Didn't specify

Study duration: __________________ ☐ Didn't specify

How many examiners? ____________________

Examiner Intra and inter-Calibration? ____________________

Part II: Subject Information

Subject age: ____________________

Number of subjects: ____________

Subject race (number/percentage if applicable):

☐ Caucasian _______________
☐ African American _______________
☐ Asian _______________
☐ Others (describe) ______________

Subject medical background:

☐ Healthy
☐ non-healthy (specify disease) ____________

Part III: Oral Health Care Delivery Method

Caries clinical examination method:

☐ Visual-tactile examination
☐ Radiograph
☐ Others (describe) ____________

Caries recording system:

☐ *dmft/s*
☐ ICDAS
☐ Others (describe) ____________

Types of prenatal oral care

☐ Visual-tactile examination
☐ Cleaning
☐ Oral health education
☐ Others (describe) _______________

Who delivered prenatal oral care?

☐ Dentist
☐ Hygienist
☐ Social worker
☐ OB doctor
☐ Primary health care physician
☐ Midwife
☐ Others (describe) _______________

Part IV: Any oral sample collected? If yes, proceed with the following:

Sample source (If applicable):

☐ Saliva: ☐ Non-stimulated ☐ Stimulated ☐ Didn't specify
☐ Plaque: ☐ Supragingival ☐ Subgingival ☐ mixed ☐ Sound surface ☐ Carious lesion
☐ Swab
☐ others (describe) ____________

Bacteria isolation and identification method:

☐ Culture (Describe type of plate agar) ____________
☐ PCR
☐ others (describe) ___________

Examined bacteria type:
☐ *S. mutans* ☐ *Lactobacillus* ☐ others (describe) ___________

Part V: Results

- Improved oral health knowledge: ☐ Yes ☐ No
- Improved oral health during pregnancy: ☐ Yes ☐ No
- Reduced dental caries in children: ☐ Yes ☐ No
- Reduced *Streptococcus mutans* carriage ☐ Yes ☐ No

Part VI: Statistical analysis results (if applicable)

☐ **Statistical analysis type: ____________________________**

Author's conclusion:

____

____

Reviewer's comments:

____

____

Appendix 3: Excluded articles after full-text review

1 Stanley, V. C. & Hurley, R. Candida precipitins in pregnant women: validity of the test systems used. *J Clin Pathol* **27**, 66-69, doi:10.1136/jcp.27.1.66 (1974).

2 el-Gally, K. A. & Shoeb, S. A. Caries incidence and aciduric microorganisms in pregnancy. *Egypt Dent J* **25**, 137-146 (1979).

3 Scheffer, P., Mautaint, C. & Dublanchet, A. [Salivary pH during pregnancy. Relationship with the presence or absence of Candida albicans. A study of 200 cases (author's transl)]. *Rev Stomatol Chir Maxillofac* **82**, 140-142 (1981).

4 Tenovuo, J., Laine, M. & Lehtonen, O. P. Salivary IgA antibodies reacting with Streptococcus mutans in relation to changes in salivary S. mutans counts: a longitudinal study in humans. *Proc Finn Dent Soc* **83**, 55-59 (1987).

5 Jonsson, R., Howland, B. E. & Bowden, G. H. Relationships between periodontal health, salivary steroids, and Bacteroides intermedius in males, pregnant and non-pregnant women. *J Dent Res* **67**, 1062-1069, doi:10.1177/00220345880670080101 (1988).

6 Raber-Durlacher, J. E., van Steenbergen, T. J., Van der Velden, U., de Graaff, J. & Abraham-Inpijn, L. Experimental gingivitis during pregnancy and post-partum: clinical, endocrinological, and microbiological aspects. *J Clin Periodontol* **21**, 549-558, doi:10.1111/j.1600-051x.1994.tb01172.x (1994).

7 Tsai, C.-C. & Chen, K.-S. A study on sex hormones in gingival crevicular fluid and black pigmented bacteria in subgingival plaque of pregnant women. *Kaohsiung Journal of Medical Sciences* **11**, 265-273 (1995).

8 Offenbacher, S. *et al.* Potential pathogenic mechanisms of periodontitis associated pregnancy complications. *Annals of periodontology / the American Academy of Periodontology* **3**, 233-250, doi:10.1902/annals.1998.3.1.233 (1998).

9 Jean-Baptiste, M. Caries prevention during pregnancy: results of 30-month study. *J Nurse Midwifery* **44**, 164-165, doi:10.1016/s0091-2182(99)00019-1 (1999).

10 Villagrán, E., Linossier, A. & Donoso, E. [Count of salivary Streptococci mutans in pregnant women of the metropolitan region of Chile: cross-sectional study]. *Rev Med Chil* **127**, 165-170 (1999).

11 Holbrook, W. P. *et al.* No link between low-grade periodontal disease and preterm birth: A pilot study in a healthy Caucasian population. *Acta Odontologica Scandinavica* **62**, 177-179, doi:10.1080/00016350410001522 (2004).

12 Urbán, E. *et al.* Distribution of anaerobic bacteria among pregnant periodontitis patients who experience preterm delivery. *Anaerobe* **12**, 52-57, doi:10.1016/j.anaerobe.2005.08.001 (2006).

13 Herrera, G. C., Pantoja, F. P., De la, M. T. L., Sanhueza, C. A. & Salazar, N. L. [Microbiologic and molecular diagnostic of cariogenic bacteria in pregnant women from the Araucania Region of Chile]. *Rev Chilena Infectol* **24**, 270-275, doi:10.4067/s0716-10182007000400002 (2007).

14 Tarannum, F. & Faizuddin, M. Effect of periodontal therapy on pregnancy outcome in women affected by periodontitis. *J Periodontol* **78**, 2095-2103, doi:10.1902/jop.2007.060388 (2007).

15 Ovalle, A. *et al.* Relationship between periodontal diseases and ascending bacterial infection with preterm delivery. *Revista Medica De Chile* **137**, 504-514 (2009).

16 Akbar, A., Ariningtyas, N. D., Krismariono, A. & Dachlan, E. G. OS002. Comparison TNF-alpha level and pathogen periodontal microorganismbetween normal pregnant women, periodontitis pregnant women, and lateonset severe preeclampsia women. *Pregnancy hypertension* **2**, 175-176, doi:10.1016/j.preghy.2012.04.004 (2012).

17 Carrillo-de-Albornoz, A., Figuero, E., Herrera, D., Cuesta, P. & Bascones-Martinez, A. Gingival changes during pregnancy: III. Impact of clinical, microbiological, immunological and socio-demographic factors on gingival inflammation. *Journal of Clinical Periodontology* **39**, 272-283, doi:10.1111/j.1600-051X.2011.01800.x (2012).
18 Chen, Z. B. *et al.* [Relationship between the preterm low birth weight infant and the periodontal pathogen bacteria in maternal saliva]. *Beijing Da Xue Xue Bao Yi Xue Ban* **44**, 29-33 (2012).
19 Sugita, N. *et al.* Immunoregulatory gene polymorphisms in Japanese women with preterm births and periodontitis. *J Reprod Immunol* **93**, 94-101, doi:10.1016/j.jri.2012.01.005 (2012).
20 Swati, P., Thomas, B., Vahab, S. A., Kapaettu, S. & Kushtagi, P. Simultaneous detection of periodontal pathogens in subgingival plaque and placenta of women with hypertension in pregnancy. *Archives of Gynecology and Obstetrics* **285**, 613-619, doi:10.1007/s00404-011-2012-9 (2012).
21 Tateishi, F. *et al.* Detection of Fusobacterium nucleatum in chorionic tissues of high-risk pregnant women. *J Clin Periodontol* **39**, 417-424, doi:10.1111/j.1600-051X.2012.01855.x (2012).
22 Ercan, E. *et al.* Evaluation of periodontal pathogens in amniotic fluid and the role of periodontal disease in pre-term birth and low birth weight. *Acta Odontol Scand* **71**, 553-559, doi:10.3109/00016357.2012.697576 (2013).
23 Usin, M. M., Tabares, S. M., Parodi, R. J. & Sembaj, A. Periodontal conditions during the pregnancy associated with periodontal pathogens. *J Investig Clin Dent* **4**, 54-59, doi:10.1111/j.2041-1626.2012.00137.x (2013).
24 Chaloupka, P., Korečko, V., Turek, J. & Merglová, V. [Oral health status of women with normal and high-risk pregnancies]. *Ceska Gynekol* **79**, 29-33 (2014).
25 Purushothama, P., Rajanna, A. K. A. & Ravi, S. B. Estimation of streptococcus mutans count in saliva of pregnant Women- A Case-Control study. *Indian Journal of Public Health Research and Development* **5**, 263-267, doi:10.5958/0976-5506.2014.00056.4 (2014).
26 Boustedt, K., Roswall, J., Dahlen, G., Dahlgren, J. & Twetman, S. Salivary microflora and mode of delivery: a prospective case control study. *Bmc Oral Health* **15**, doi:10.1186/s12903-015-0142-3 (2015).
27 Gomez-Arango, L. F. *et al.* Contributions of the maternal oral and gut microbiome to placental microbial colonization in overweight and obese pregnant women. *Sci Rep* **7**, 2860, doi:10.1038/s41598-017-03066-4 (2017).
28 da Silva, C. B. *et al.* Streptococcus mutans detection in saliva and colostrum samples. *Einstein-Sao Paulo* **17**, doi:10.31744/einstein_journal/2019AO4515 (2019).
29 Emory, U. Biobehavioral Determinants of the Microbiome and Preterm Birth in Black Women. *European Nucleotide Archive* (2019).
30 Mohr, S. *et al.* Systemic Inflammation in Pregnant Women With Periodontitis and Preterm Prelabor Rupture of Membranes: A Prospective Case-Control Study. *Frontiers in Immunology* **10**, doi:10.3389/fimmu.2019.02624 (2019).
31 de Oliveira, M. C. *et al.* Microbiological findings of the maternal periodontitis associated to low birthweight. *Einstein-Sao Paulo* **18**, doi:10.31744/einstein_journal/2020AO4209 (2020).
32 Thakur, R. K. *et al.* Influence of Periodontal Infection as a Possible Risk Factor for Preterm Low Birth Weight. *J Pharm Bioallied Sci* **12**, S613-s618, doi:10.4103/jpbs.JPBS_73_20 (2020).

Appendix 4: Quality of evidence and strength of recommendation followed by GRADE

	Quality of evidence	Strength of Recommendation
Brambilla et al, 1998	⊕⊕⊕◯<br>Moderate	↑↑<br>Strong for an intervention
Mitchell-Lewis et al, 2001	⊕⊕◯◯<br>Low	↑?<br>Weak for an intervention
Offenbacher et al., 2006	⊕⊕⊕◯<br>Moderate	↑↑<br>Strong for an intervention
Novak et al, 2008	⊕⊕⊕◯<br>Moderate	↑↑<br>Strong for an intervention
Volpato et al, 2008	⊕⊕◯◯<br>Low	↑?<br>Weak for an intervention
Jaramillo et al, 2012	⊕⊕◯◯<br>Low	↑↑<br>Strong for an intervention
Asad et al, 2018	⊕⊕◯◯<br>Low	↑?<br>Weak for an intervention
Escalante-Medina et al, 2019	⊕⊕◯◯<br>Low	↑?<br>Weak for an intervention
